# A tablet-based intervention study to alleviate cognitive and psychological symptoms in patients with post-Covid-19 condition

**DOI:** 10.3389/fpsyg.2025.1582742

**Published:** 2025-08-25

**Authors:** Manuel Leitner, Lucas Paletta, Manuel Leal-Garcia, Maria Fellner, Marisa Koini

**Affiliations:** ^1^Department of Neurology, Medical University of Graz, Graz, Austria; ^2^Department of Medical Psychology, Psychosomatics and Psychotherapeutic Medicine, Medical University of Graz, Austria; ^3^Joanneum Research, Graz, Austria; ^4^Probando GmbH, Graz, Austria; ^5^DigitAAL Life GmbH, Graz, Austria

**Keywords:** post-Covid-19 condition, long-Covid, tablet-based intervention, cognition, subjective cognitive complaints, depressive symptoms, mental health, psychological well-being

## Abstract

**Background:**

Cognitive impairment and psychological complaints are among the most common consequences for patients suffering from Post-Covid-19 condition (PCC). As there are limited training options available, this study examined a longitudinal tablet-based training program addressing cognitive and psychological symptoms.

**Methods:**

Forty individuals aged between 36 and 71 years (*M* = 49.85, *SD* = 8.63; 80% female) were randomly assigned to either an intervention group (*n* = 20) or a waitlist control group (*n* = 20). The intervention group received a three-month tablet-based training program involving cognitive exercises, relaxation techniques, and physiotherapy exercises. Additionally, both groups underwent a thorough neuropsychological assessment (attention, memory, executive functions, word fluency, subjective cognitive complaints, fatigue, depression, anxiety, and quality of life) before the training, after 3 months of training, and after 6 months in order to assess long-term effects.

**Results:**

Pre-post comparisons revealed that individuals assigned to the intervention group (*n* = 18 after dropout), as compared to the control group (*n* = 16 after dropout), showed a reduction in subjective cognitive complaints (*p* < 0.001) as well as in depressive symptoms (*p* < 0.001). Additionally, their MoCA Memory Index Score remained stable (*p* = 0.496), while it declined significantly in the wait-list control group (*p* = 0.008). However, the training had no effect on the other domains assessed and not all training-related effects were stable over time. Finally, a higher number of post-Covid symptoms was negatively correlated with attention and memory capabilities (all *p* < 0.05), with a longer disease duration further amplifying the negative impact of post-Covid symptoms on memory performance.

**Conclusion:**

Tablet-based training programs can help improve subjective complaints, depressive symptoms, and memory and may serve as an additional therapy option. Further studies are needed to investigate the stability of these effects.

## Introduction

Since the onset of the coronavirus pandemic in March 2020, the World Health Organization (WHO) reported more than 777 million confirmed cases of Covid-19 and over 7 million deaths until December 2024 (World Health Organization [WHO], 2024). The majority of individuals infected with SARS-CoV-2 are dealing with symptoms such as fever, cough, and fatigue during their acute illness (Carfi et al., 2020; Grant et al., 2020; Ziauddeen et al., 2022), which typically lasts up to 14 days after the symptom onset (O’Mahoney et al., 2023). However, an increasing number of individuals report persistent or newly emerging symptoms after their initial infection subsided (Carfi et al., 2020; Fernández-de-Las-Peñas et al., 2021; Sykes et al., 2021; van Kessel et al., 2022). These symptoms can be grouped under the umbrella term “Long-Covid or Post-Covid-19 condition (PCC).” PCC is defined as the “continuation or development of new symptoms 3 months after the initial SARS-CoV-2 infection, with these symptoms lasting for at least 2 months with no other explanation” (Lippi et al., 2023; World Health Organization [WHO], 2022). However, as there are several definitions used for this complex condition (Fernández-de-Las-Peñas et al., 2023), PCC remains vaguely defined and no agreement on a standardized definition has been made yet (Munblit et al., 2022). Consequently, this resulted in great heterogeneity with respect to the PCC definitions used in interventional studies (Fernández-de-Las-Peñas et al., 2022; Haslam et al., 2023). In this study, we follow the aforementioned and universally acknowledged definition of PCC (long-Covid), as suggested by the WHO (Lippi et al., 2023; World Health Organization [WHO], 2022).

The prevalence of post-Covid will remain high (Boufidou et al., 2023) and studies indicate that globally approximately 10%–20% (Altmann et al., 2023; Ballering et al., 2022; Davis et al., 2023; World Health Organization [WHO], 2022) of individuals are affected by PCC, with about 18%–22% particularly experiencing cognitive symptoms (Ceban et al., 2022; Han et al., 2022). In addition, several risk factors have been identified that include, for instance, female sex (Fernández-de-Las-Peñas et al., 2022; Hedberg et al., 2023; Tene et al., 2023), older age (Abdelrahman et al., 2021; Thompson et al., 2022; Bonfim et al., 2024), smoking (Barthélémy et al., 2022; Tene et al., 2023; Tsampasian et al., 2023), preexisting comorbidities (Subramanian et al., 2022; Tsampasian et al., 2023) and an elevated body mass index (Subramanian et al., 2022; Sudre et al., 2021; Thompson et al., 2022; Tsampasian et al., 2023). In contrast, being vaccinated prior to a SARS-CoV-2 infection was shown to reduce the risk of developing PCC (Byambasuren et al., 2023; Krishna et al., 2023; Wong et al., 2023), suggesting that vaccination might be a protective factor against the development of persistent Covid-19 symptoms.

It is important to note that persistent symptoms can occur regardless of whether individuals were hospitalized during their acute illness or not (O’Mahoney et al., 2023; Leitner et al., 2024; Nalbandian et al., 2023), that means regardless of their illness severity (Di Gennaro et al., 2023; Vanichkachorn et al., 2021). Until today, over 200 different symptoms have been identified (Davis et al., 2023), including fatigue (Scardua-Silva et al., 2024; Torrell et al., 2024; Zhang et al., 2024), cough, or loss of sense of smell/taste (Byambasuren et al., 2023). Additionally, Covid-19 infection can cause long-term effects on the cognitive function and psychological well-being of those affected (Gonzalez-Fernandez and Huang, 2023; Jaywant et al., 2024; Möller et al., 2023), which leads to impairments such as brain fog (McWhirter et al., 2023; Lanz-Luces et al., 2022; Jennings et al., 2022), concentration difficulties (Ziauddeen et al., 2022; Ruzicka et al., 2024; Kim et al., 2023), attention disorders (Cipolli et al., 2023; Kozik et al., 2023; Guillén et al., 2024), executive dysfunction (Dacosta-Aguayo et al., 2024; Hampshire et al., 2024; Godoy-González et al., 2023), memory problems (Hampshire et al., 2024; Fleischer et al., 2022; Martin et al., 2024), anxiety (Seighali et al., 2024; Wong et al., 2023; Goodman et al., 2023) or depression (Seighali et al., 2024; McLaughlin et al., 2023; Kim et al., 2023). These symptoms may last for several months or years (Herrera et al., 2023) and manifest not only as objective deficits but also as subjective cognitive complaints (Miskowiak et al., 2021), causing substantial burden for those affected.

Although both the infection rates and the incidence of PCC remain high (Di Gennaro et al., 2023), general treatment options are limited (Davis et al., 2023; Koczulla et al., 2021; Mueller et al., 2023; Veronese et al., 2022), especially concerning the treatment of cognitive deficits (Yong, 2021). Notably, one in five individuals will exhibit cognitive impairments three or more months after receiving a Covid-19 diagnosis (Ceban et al., 2022; Möller et al., 2023). A previous study by Jebrini et al. (2024) used cognitive training and group psychotherapy to increase verbal memory and visuo-spatial construction skills in patients with long-Covid (Jebrini et al., 2024). Further study protocols have been designed to improve cognitive performance in patients through either brain stimulation-assisted cognitive training (Thams et al., 2022) or Goal Management Training (Hagen et al., 2022). However, fatigue represents a major challenge, as it was reported as one of the most common long-Covid symptoms (Joli et al., 2022; Leitner et al., 2024), limiting the opportunity to take part in such rehabilitation programs. Finally, the rehabilitation progress of patients is complicated by the heterogeneous clinical picture of this disease, which highlights the need for a tailored and multidisciplinary rehabilitation approach (Nice, 2020; Krishna et al., 2023; Nurek et al., 2021).

One opportunity to address cognitive deficits involves the use of tablets and computers. Computerized cognitive training programs have been tested in various populations, including patients with diabetes (Bahar-Fuchs et al., 2020), Parkinson’s disease (Gavelin et al., 2022), or stroke (Zhou et al., 2022) and can often be applied in familiar environments (e.g., one’s own home). A pilot study conducted among individuals with self-reported cognitive dysfunction for more than 3 months after an infection with the coronavirus suggested that computerized cognitive training may be effective in improving cognitive impairments (Duñabeitia et al., 2023). However, the authors concluded that future studies including a control group are necessary to draw reliable conclusions. Similar results were found in a case-control study among seventy-three Covid-19 survivors with cognitive impairment (Palladini et al., 2023). The authors of the study used a cognitive remediation therapy (CRT) which showed positive results on cognitive functioning. These results support CRT as an effective treatment option targeting cognitive impairments in patients suffering from PCC (Palladini et al., 2023).

In general, only a small number of therapeutic intervention options are available for the treatment of cognitive and mental deficits in patients with long-Covid (Davis et al., 2023; Koczulla et al., 2021; Mueller et al., 2023; Veronese et al., 2022). Therefore, this study aims to improve these symptoms by using a three-month tablet-based training program that includes a variety of cognitive exercises (e.g., to train attention, memory and executive functions) as well as relaxation and physiotherapy exercises (see “Tablet-based intervention” in the Methods section). These trainings offer the advantage of being conducted from home (i.e., location independent) and allow individuals to practice at their own pace (self-paced). In addition, long travel times, which could be challenging for some patients, can be eliminated.

**Primary hypotheses:** We hypothesize that participating in our training program alleviates cognitive symptoms associated with PCC in various domains, such as memory, attention, or executive functions. In addition, we hypothesize that the intervention will reduce subjective cognitive complaints and psychological symptoms, including fatigue and negative emotions such as anxiety and depression, as well as improve the quality of life of those affected by PCC. **Exploratory analyses:** In exploratory analyses, we further investigate the association between post-Covid symptom count and cognition, and the impact of disease duration on this correlation.

## Materials and methods

### Recruitment, participants and procedure

**FIGURE 1:**
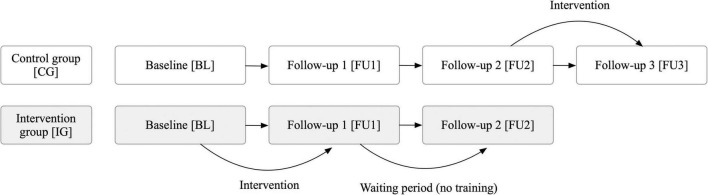
Study procedure to evaluate the effect of a three-month tablet-based intervention in patients with post-Covid-19 condition. Thorough neuropsychological assessments were conducted at each time point (BL, FU1, FU2, FU3), i.e., every 3 months. A waiting period was included to assess the stability of long-term effects.

The recruitment was carried out by the company “Probando GmbH,” which recruited patients through newspaper articles and online posts. Forty-two patients with self-reported symptoms of PCC (i.e., persistent symptoms or newly emerged symptoms after the resolution of an acute Covid-19 infection) were invited to undergo a comprehensive neuropsychological assessment as well as structural and functional MRI at the Medical University of Graz, Austria between October 2022 and November 2023. Inclusion criteria were (a) ongoing or newly developed symptoms 3 months after a positive Covid-19 infection, (b) with these symptoms lasting for at least 2 months with no other explanation (World Health Organization [WHO], 2022), and (c) symptoms leading to either a new health impairment (self-report) or deterioration of a pre-existing disease (self-report), and (d) none of the following pre-existing diseases: dementia, multiple sclerosis, Parkinson’s disease, stroke, and (e) no participation on any other pharmacological or psychological training study aiming to improve cognitive or psychological complaints. Due to significant impairment (high Post-COVID-19 functional status (Klok et al., 2020) combined with high levels of fatigue), two individuals were excluded from further study participation although initially meeting inclusion criteria (after extensive discussion, these individuals were deemed unable to adequately complete the training over the 3-month duration). The remaining 40 participants were randomly (block randomization, block size = 6) assigned to either an intervention (*n* = 20) or a wait-list control group (*n* = 20) by MK after completing a baseline (BL) testing. The person who conducted the cognitive assessment (ML) was blinded to the subjects’ group allocation (intervention group vs. control group). However, due to the study design, blinding of the participants was not possible. Therefore, participants knew whether they were receiving the intervention or not. Individuals assigned to the intervention group received a free tablet-based training program and had three on-site neuropsychological examinations with 3 months between each examination period to assess both a post-training effect [BL to follow up 1 (FU1)] as well as the stability of this effect (FU1 to FU2). Those who were assigned to the control group received no training or treatment as usual (since no validated treatment existed at the time the study was conducted) for the first three assessments (BL-FU1-FU2) but they received the same training after a six-month waiting period in order to assess their post-training effect (FU2-FU3) as well (Figure 1). Finally, in exploratory analyses, both groups were combined to assess pre-post effects in a larger sample.

**FIGURE 2:**
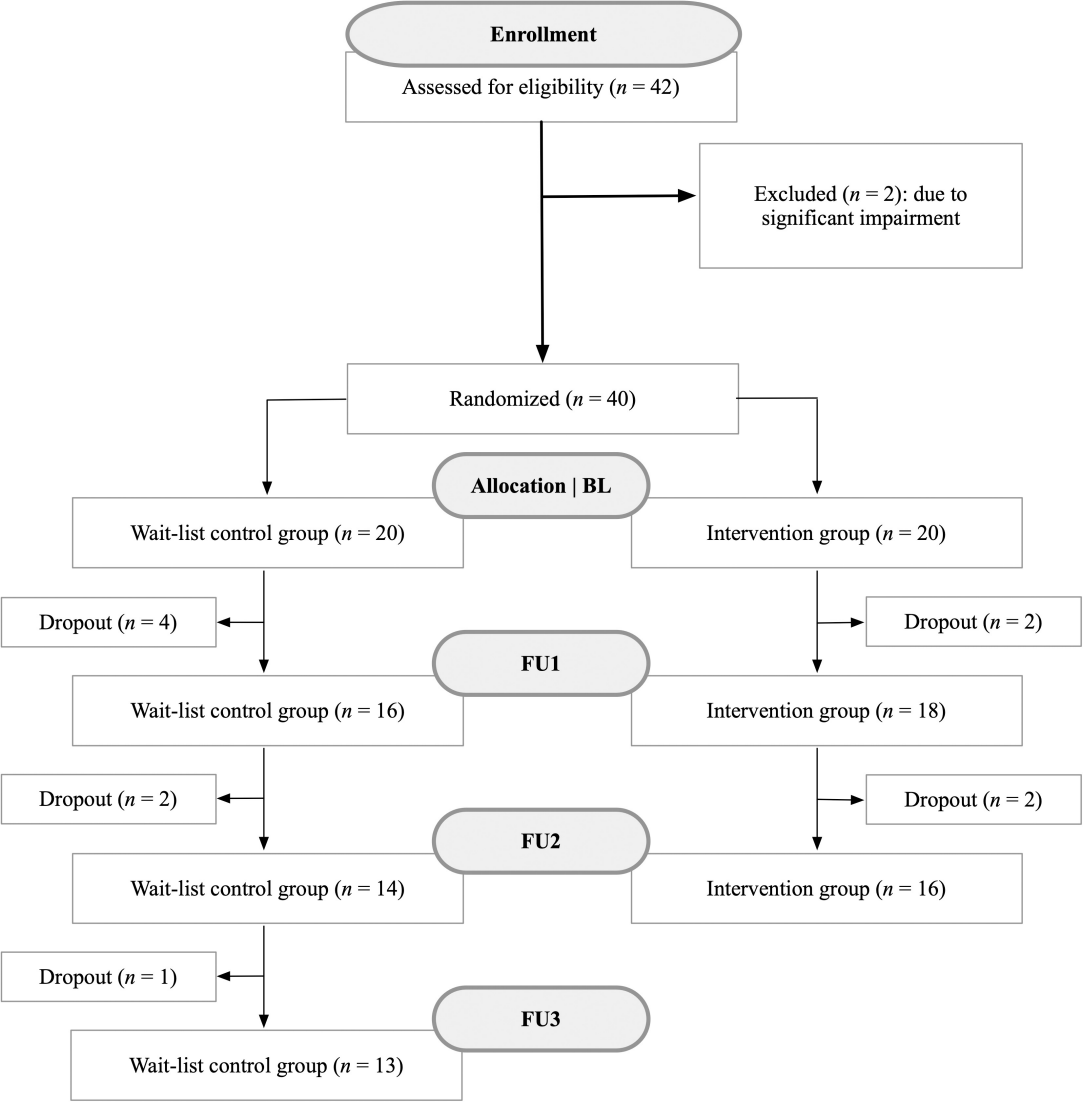
Flow chart illustrating the recruitment process of patients with post-Covid-19 condition and associated dropouts between October 2022 and November 2023. This study utilized a longitudinal design to observe changes over time. Reasons for dropouts included, for instance, loss of motivation in the training program or rejection of any further thorough neuropsychological assessments.

Due to the longitudinal study design, there were occasional dropouts during the examination period (Figure 2). All dropouts occurred due to patients who terminated their participation in the study. Participants who ceased participation did not differ from those who continued the study in sex, fatigue, or cognition (*p* > 0.05). At each time point, a comprehensive neuropsychological test battery (see assessments section) was administered along with questionnaires to assess cognition and psychological parameters such as depression or anxiety. We additionally gathered data on symptoms during the acute Covid-19 illness as well as still persistent symptoms at each visit. Participants were explicitly asked to provide only symptoms that have not occurred prior to the illness (e.g., if they had memory problems before their Covid-19 infection, they were asked to not report them as a consequence of the infection). The entire study procedure took about 2 h at each assessment.

### Assessment

We collected information on the following variables: age, sex, highest level of education completed, marital status, height, weight, date of Covid-19 diagnosis, duration of acute infection, vaccination status, initial symptoms, current symptoms, hospitalization, functional status [Post-COVID-19 Functional status scale (Klok et al., 2020)], pre-existing conditions, as well as psychiatric disorders. In addition, we administered a comprehensive neuropsychological assessment battery and questionnaires on fatigue, depression, anxiety, subjective cognitive complaints and quality of life to all participants. The following domains were assessed:

#### Global cognitive impairment

We used the Montreal Cognitive Assessment (MoCA) as a cognitive screening test, aiming to detect global cognitive deficits (Nasreddine et al., 2005). Higher scores indicate better cognitive performance. The memory index score (MIS) was used to assess memory performance in a simple memory task (recollection of five previously read words).

#### Attention

To assess the attentional performance of participants, the subtests “Digit Span Forward” and “Digit Span Backward” from the German version of the Neuropsychological Assessment Battery (NAB) were used. Higher values indicate better attentional performance (Petermann et al., 2013). Additionally, the Trail Making Test A (TMT-A) was used to measure attention. A shorter processing time indicates better attentional performance (Reitan, 1958).

#### Executive function

The subtests “Planning” and “Categories” from the NAB were utilized to assess executive functions of patients. Higher scores represent a better performance in executive tasks (Petermann et al., 2013). Additionally, the Trail Making Test B (TMT-B) can be utilized as a tool for assessing executive functions (Arbuthnott and Frank, 2000). A shorter processing time reflects better executive functions.

#### Memory

For the assessment of memory performance, the subtest “Word List Learning” of the NAB was administered. Participants are required to remember as many words as possible out of a word list (12 words). Higher scores indicate a better memory performance (Petermann et al., 2013).

#### Word fluency

We used the Regensburger Word Fluency Test (RWT) to assess formal-lexical and semantic word fluency. In this task, individuals are required to verbally generate as many words as possible either starting with a specific letter (formal-lexical) or belonging to a particular category (semantic) within 2 min. Higher scores correspond to better performance in word fluency (Aschenbrenner et al., 2001).

#### Subjective cognitive complaints

To assess subjective cognitive complaints (SCC), a translated version of the Questionnaire de Plainte Cognitive (Thomas-Antérion et al., 2004) was utilized. This questionnaire had already been translated into English (Markova et al., 2017) and was further translated (and back-translated) into German by independent translators. Participants are asked to respond with “Yes” or “No” and indicate whether they have perceived any changes in themselves in the last 6 months (e.g., “Have you experienced any memory change during the last 6 months?”). The maximum score is 10 points. A score of 3 or higher is considered clinically significant (Thomas-Antérion et al., 2004).

#### Fatigue

The German version of the Fatigue Impact Scale (FIS-D) was used to assess fatigue (Häuser et al., 2003). It consists of 40 questions, answered on a 5-point scale from “never” (0) to “very often” (4). A higher total score corresponds to a higher level of fatigue.

#### Depression and anxiety

The extent of depression was assessed using two questionnaires, namely the General Depression Scale in its 20-item long form (Hautzinger et al., 2012) and the German version of the Hospital Anxiety and Depression Scale (Herrmann-Lingen et al., 2018). The General Depression Scale (ADS, Allgemeine Depressionsskala) is a self-assessment tool used to evaluate the impact of depressive symptoms experienced in the past week. While completing the HADS, patients need to report the extent of anxiety and depressive symptoms experienced in the past week. Higher scores indicate a higher level of depression and/or anxiety.

#### Quality of life

The German version of the WHOQoL-BREF (World Health Organization Quality of Life) in its short form (26 items) was used to assess patients’ quality of life (Angermeyer et al., 2000) during the past 2 weeks. The subscales for physical and psychological well-being were used for analyses.

### Tablet-based intervention

The program was developed by DigitAAL Life GmbH, a company that designed cognitive training programs for different populations, such as patients with Alzheimer’s disease and those with long-Covid/post-Covid-19 condition. Before the patients were provided with the tablet and the installed training, each patient received individual on-site instruction, during which all participants became familiar with the technology and the exercises. Using the tablet required no specific technical knowledge. The intervention combined relaxation exercises [Jacobson muscle relaxation (Jacobson, 1938)], physiotherapy exercises and cognitive training. As the training was conducted asynchronously and from home, we were able to offer a location-independent training program, allowing patients to train as often as they wanted and at flexible times. The cognitive part included tasks such as remembering and recalling sequences (visual memory; e.g., remembering a sequence of fields and then clicking them in the same order), attention/reaction time exercises (e.g., participants had to click on the screen as fast as possible whenever they saw a number appear anywhere on it, in order to train their sustained attention), calculation tasks (e.g., solving mathematical calculation tasks), exercises to train executive functions (e.g., exercises with inhibition tasks), as well as playful activities such as the game “memory” or quiz tasks (to train short- and long-term memory). In addition, relaxation exercises, such as progressive muscle relaxation (PMR) according to Jacobson, were integrated into the training, and simple physiotherapy exercises were performed. These included, for example, exercises with balls or resistance bands, in which patients had to carry out physical exercises simultaneously with cognitive tasks. Both the physiotherapy and relaxation exercises were delivered to the patients via videos, in which they were instructed to follow the exercises. In PMR, for instance, patients learned to tense and then release the muscles in different areas of their body, which is known to foster relaxation. It was recommended to train at least three times a week for at least 30 min each session, with more frequent training being encouraged. Additionally, various difficulty levels were implemented to adapt the tasks to the cognitive abilities of the participants. Patients were contacted by phone every 2 weeks to ensure they continued training and to receive feedback. They could contact the study authors at any time if problems occurred.

### Sample size calculation

Prior to the commencement of the study, a power analysis with G*Power was conducted to calculate the required sample size for the study design (Faul et al., 2007). Two meta-analyses investigating the effects of computerized cognitive training programs on cognitive impairment (Hu et al., 2021; Hill et al., 2017), as well as a study examining personalized computerized training for cognitive dysfunction after Covid-19 (Duñabeitia et al., 2023), found at least medium-sized effects with respect to cognitive outcomes. As the primary focus of our study was to compare both groups (CG, IG) across three time points (BL, FU1, FU2), a power analysis for a 2 × 3 analysis of variance (ANOVA) was performed. 28 individuals (14 per group) are needed to detect a medium-sized effect ($\eta_{p}^{2}$ = 0.06) with 80% power (1- β) at a significance level of α = 0.05. Due to potential dropouts, we exceeded the calculated minimum sample size with a total of *n* = 40 individuals (*n* = 20 per group).

### Statistical analysis

Statistical analyses were performed with SPSS (Version 29.0). The figures were created using RStudio (R version 4.2.2). For all variables, normality was checked by using Shapiro-Wilk tests as well as histograms and Q-Q-plots. To examine the effect of the tablet-training on the improvement in cognitive and psychological symptoms, 2 × 3 analyses of variance were conducted. Hence, group (levels: control group, intervention group) represented the between-subjects factor and time (levels: BL, FU1, FU2) was the within-subjects factor. To statistically control for potential confounders such as age, sex, and education, these were included as covariates in the analyses, if the assumptions were met (1. homogeneity of regression slopes, 2. no group differences in the covariates). If an assumption was violated, the covariate was dropped from the model. Since the interaction effects of the individual 2 × 3 analyses may not be significant, even though a difference from baseline to follow-up 1 in the intervention group (compared to the control group) might be present, additional 2 × 2 analyses of variance (with covariates) were calculated. This could especially be the case if the effect (e.g., improvement in cognition in the intervention group from baseline to follow-up 1) declines until the next visit (follow-up 2), or when the control group shows spontaneous improvement between the time points. Significant interaction effects were further examined using *post hoc* tests to assess whether the groups differed at specific time points and whether changes were observable within each group.

The assumption of homogeneous variances between the groups was tested by using Levene’s tests for the respective outcome variable at each time-point. To test whether the observed covariance matrices of the respective dependent variables are equal across groups, the Box’s M test was used. Mauchly’s test of sphericity was used to assess the assumption of sphericity in the 2 × 3 analyses. In case of a violation of sphericity, Greenhouse-Geisser correction was applied. Bonferroni correction was applied to all *p*-values.

In further exploratory analyses, individuals from the waitlist control group, after completing the training as well, were merged with the original intervention group to form a combined intervention group (including all individuals who successfully completed the training), and pre-post comparisons were then conducted (pre values for intervention group: baseline; pre values for control group: follow-up 2; post values for intervention group: follow-up 1; post values for control group: follow-up 3). Hence, we performed a pre-post comparison of all individuals who eventually received the training. This approach was chosen to increase statistical power. Paired *t*-tests for each outcome were calculated for this purpose. To avoid bias in the results (e.g., significant reduction in pre-post comparison due to familiarity with the test material or spontaneous improvements), results were only interpreted if there were no familiarity effects or spontaneous changes in the original control group. Therefore, and due to the small sample size when considering only the original control group, Friedman tests were performed for the data of the control group. Finally, significant *t*-tests were interpreted only if the original control group did not exhibit a significant change over time. This approach helped us rule out spontaneous symptom improvements or familiarity effects as explanations for the significant changes in the paired *t*-tests.

For correlation analyses (e.g., analyzing the relationship between the number of post-Covid symptoms and cognition), Pearson correlation coefficients (controlled for age, sex, and education) were computed. In addition, we examined whether patients’ disease duration moderated the association between post-Covid symptom count and cognition. Therefore, we performed moderation analyses by using the PROCESS SPSS Makro [Version 4.1, (Hayes, 2022)] which is available online^1^. To enhance the interpretation of main effects, variables that define the product term/interaction term (i.e., the predictor and moderator) were mean centered prior to the analyses. Additionally, moderation analyses were controlled for age, sex, and education. To counteract an alpha error inflation due to heteroscedasticity, robust standard errors of type HC3 (Davidson-MacKinnon) were used. Significant results were plotted for three specific values of the moderator (−1 *SD*, *M*, + 1 *SD*).

Finally, to assess the prevalence of cognitive impairments and psychological symptoms, we used the criteria outlined in the specific test manuals. Values more than one standard deviation below the mean (*T* < 40) and percentile ranks below 16 were considered indicative of impairment.

### Ethical considerations

This study received approval from the ethics committee of the Medical University of Graz (34-206 ex 21/22). Study recruitment was carried out through advertisements in newspapers and social networks, managed by the company “Probando GmbH.” All eligible patients provided written informed consent for data recording and agreed to the study procedures. All data were pseudonymized (assignment of a subject code) and only the authors of the manuscript have access to the subject code. Hence, no identification of individual participants is possible for others. Participants had the right to withdraw from the study at any time without providing reasons, and without any disadvantage to them. In general, patients did not receive compensation for participating in the study; however, their travel expenses to and from the study site (Medical University of Graz) were reimbursed.

## Results

**FIGURE 3:**
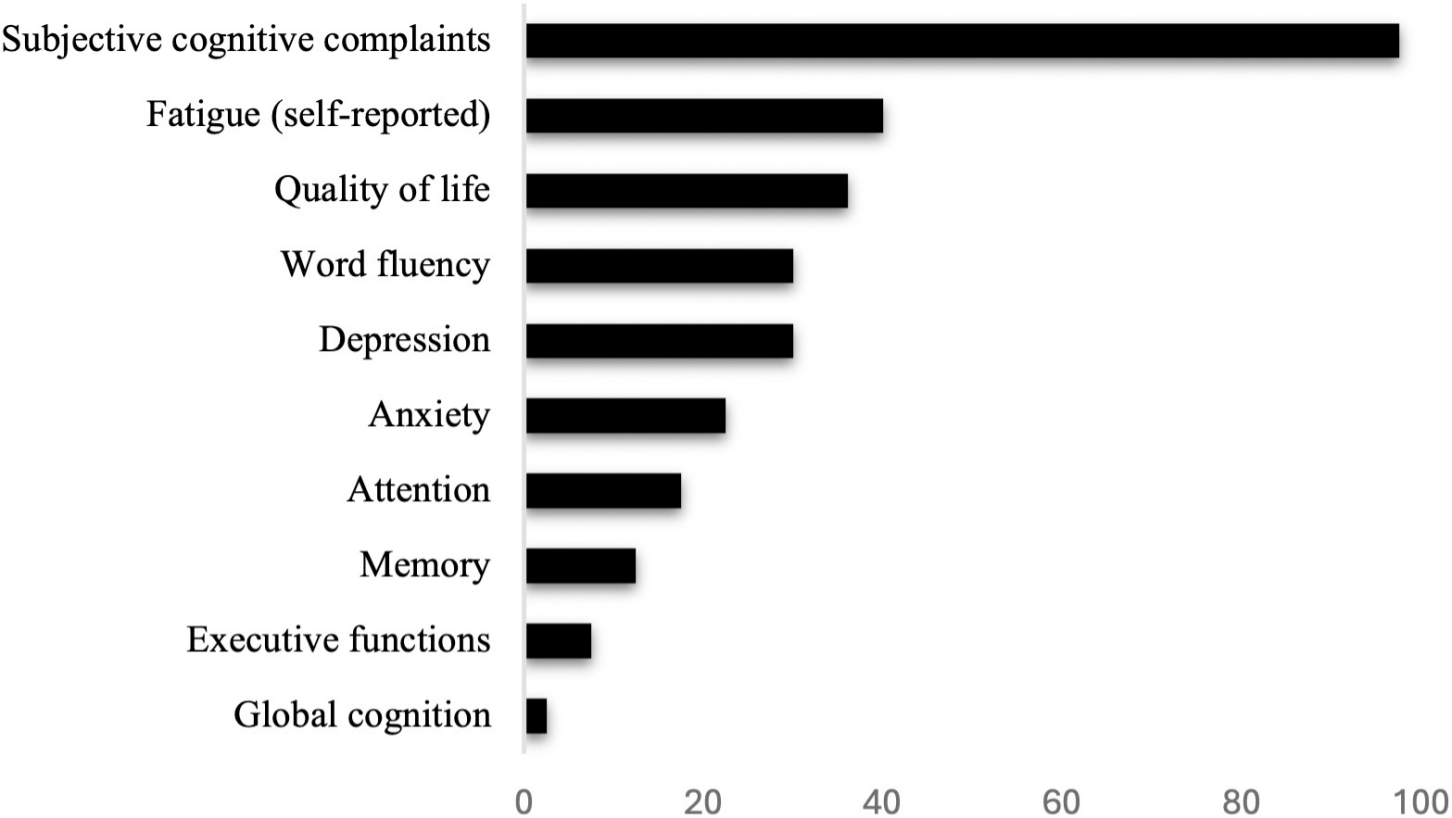
Percent of cognitive and psychological impairments of post-Covid-19 patients at baseline, evaluated according to the criteria outlined in the respective manuals or self-report (fatigue).

The final sample consisted of 40 eligible individuals (80.0% female) aged between 36 and 71 years (*M* = 49.85, *SD* = 8.63). The majority held a university degree (27.5%), completed a general higher secondary school (17.5%) or middle school without a high school diploma (15.0%). All patients met the diagnosis criteria for PCC (Lippi et al., 2023; World Health Organization [WHO], 2022). A detailed description of participants’ demographic and disease-related characteristics at baseline is provided in Table 1. No significant differences between the control and intervention group were observable at baseline. An overview of symptoms during the acute illness phase and at the different visits is provided in Supplementary Table 1. In addition, the frequency of cognitive and mental impairments for all participants at baseline as assessed by means of the test manuals’ norm scores is presented in Figure 3.

**TABLE 1:** Baseline demographic and clinical characteristics of post-Covid-19 patients assigned to the control group (CG) and intervention group (IG).

Parameter	Total (*n* = 40)		CG (*n* = 20)		IG (*n* = 20)		Difference
	*M*	*SD*	*M*	*SD*	*M*	*SD*	
Age [years]	49.85	8.63	50.15	9.64	49.55	7.73	*M*_diff_ = 0.60, *p* = 0.829
Height [cm]	167.65	7.49	167.50	7.82	167.80	7.35	*M*_diff_ = −0.30, *p* = 0.901
Weight [kg]	79.54	15.05	80.06	13.71	79.02	16.62	*M*_diff_ = 1.04, *p* = 0.830
BMI [kg/m^2^]	28.29	5.09	28.61	5.06	27.98	5.24	*M*_diff_ = 0.63, *p* = 0.701
	***Mdn***		***Mdn***	***MR***	***Mdn***	***MR***	
Education [years]	13.25		13.25	19.08	13.50	21.93	*U* = 228.50, *p* = 0.436
Disease duration [m]	20.60		22.74	22.43	15.61	18.58	*U* = 161.50, *p* = 0.298
Duration acute infection	1 [6–10 days]		1	18.93	1.50	22.08	*U* = 168.50, *p* = 0.379
Functional status	2		2	23.65	0	17.35	*U* = 137.00, *p* = 0.052
	***Count***	***%***	***Count***	***%***	***Count***	***%***	
Sex [female]	32	80.0	16	80.0	16	80.0	χ(df = 1) = 0.00, *p* = 1.000
Vaccination*^a^* [yes]	20	50.0	10	50.0	10	50.0	χ(df = 1) = 0.00, *p* = 1.000
Hospitalization [yes]	2	5.0	0	0.0	2	10.0	Fisher’s exact test: *p* = *0.487*
Pre-existing condition [yes]	24	60.0	15	75.0	9	45.0	χ(df = 1) = 3.75, *p* = 0.053

^a^Vaccination before infection. Significance tests compare the control group (CG) against the intervention group (IG). m, months; *M*, mean; *SD*, standard deviation; *Mdn*, median; *MR*, mean rank.

### Training evaluation

In the 2 × 3 ANCOVAs, we observed a significant group x time interaction only for the digit span forward task of the Neuropsychological Assessment Battery (NAB). We therefore performed Bonferroni-corrected follow-up analyses, showing that the groups differed already at baseline (*p* = 0.016), but not at FU1 (*p* = 0.757) and FU2 (*p* = 0.272). Therefore, there was neither a significant improvement in the IG from BL to FU1 (*p* = 0.345) nor from FU1 to FU2 (*p* = 1.000), nor in the CG (BL-FU1: *p* = 0.593, FU1 to FU2: *p* = 0.313). The results of all analyses are presented in Supplementary Table 2.

As a non-significant interaction effect may occur in the 2 × 3 analyses even if differences between groups from baseline to follow-up 1 are observable, we subsequently calculated 2 × 2 ANCOVAs. These analyses yielded significant group x time interaction effects in the following tests: digit span forward task (*p* = 0.032), MoCA memory index score (*p* = 0.017), subjective cognitive complaints (*p* = 0.022), and depression (ADS-L; *p* = 0.025). No significant effects (i.e., no significant differences in the slopes of the groups from BL to FU1) were observed for the other domains tested (*p* > 0.05). The results are summarized in Table 2.

**TABLE 2:** Results of the 2 × 2 ANCOVAs. Presented is the significance of the group (2 levels: control group, intervention group) × time (2 levels: BL, FU1) interaction effect to evaluate the efficacy of a three-month tablet-based training program for post-Covid-19 patients.

Domain	Subtest	*n*	*F*	df	Error df	*p*	$\eta_{p}^{2}$
Attention	Digit span forward^a^	34	5.10	1	29	**0.032**	0.150
	Digit span backward^b^	34	0.00	1	30	0.970	0.000
	TMT-A^c^	34	0.12	1	30	0.731	0.004
Executive function	Planning^b^	34	3.74	1	30	0.062	0.111
	Categories^a^	34	2.12	1	29	0.156	0.068
	TMT-B^d^	34	0.10	1	30	0.758	0.003
Memory	Immediate recall (A)^a^	34	0.19	1	29	0.669	0.006
	Immediate recall (B)^a^	34	2.93	1	29	0.098	0.092
	Short-delayed recall^a^	34	0.24	1	29	0.630	0.008
	Long-delayed recall^a^	34	0.02	1	29	0.890	0.001
Word fluency	Formal-lexical^a^	34	0.71	1	29	0.406	0.024
	Semantic^a^	34	2.05	1	29	0.163	0.066
Global cognition	MoCA^a^	34	2.16	1	29	0.153	0.069
	MoCA Memory Index^a^	34	6.40	1	29	**0.017**	0.181
Subjective cognitive complaints	FSKB [QPC]^a^	34	5.83	1	29	**0.022**	0.167
Fatigue	FISD total^a^	31	0.06	1	26	0.810	0.002
Depression	ADSL^b^	34	5.57	1	30	**0.025**	0.157
	HADS-D^b^	34	0.98	1	30	0.331	0.031
Anxiety	HADS-A^a^	34	1.07	1	29	0.311	0.035
Quality of life	WHOQoL-Physical^a^	34	1.65	1	29	0.209	0.054
	WHOQoL-Psychological^b^	34	0.14	1	30	0.710	0.005

^a^all covariates included as assumptions were met,

^b^education excluded as a covariate,

^c^sex excluded as a covariate,

^d^age excluded as a covariate. Bonferroni correction was applied to all follow-up analyses. Significant results are highlighted in bold.

Regarding the digit span forward task, both groups did differ significantly at baseline (*M*_CG.adj_ = 8.50, *SE*_CG_ = 0.44, *M*_IG.adj_ = 6.94, *SE*_IG_ = 0.41; *p* = 0.015), but not at FU1 (*M*_CG.adj_ = 7.77, *SE*_CG_ = 0.42, *M*_IG.adj_ = 7.65, *SE*_IG_ = 0.40; *p* = 0.847). However, neither the CG (*p* = 0.121) nor the IG (*p* = 0.113) significantly increased their ability in the task.

**FIGURE 4:**
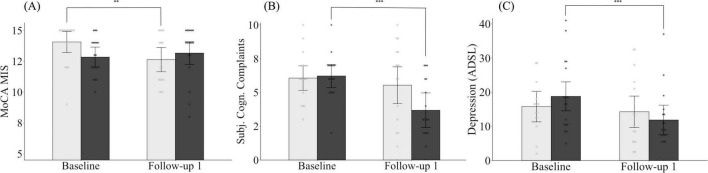
Changes in MoCA memory index score **(A)**, subjective cognitive complaints **(B)** and depression **(C)** for both the intervention and control group from baseline to follow-up 1. Light gray bars, control group; dark gray bars, intervention group. MoCA MIS, Montreal Cognitive Assessment Memory Index Score, ADSL, Allgemeine Depressionsskala (General Depression Scale). Means are adjusted for covariates (age, sex, and/or years of education). Error bars represent 95% confidence intervals (CI). *p < 0.05, **p < 0.01, ***p < 0.001.

There was no significant difference between the CG and the IG in terms of their MoCA memory index score at baseline (*M*_CG.adj_ = 14.06, *SE*_CG_ = 0.44, *M*_IG.adj_ = 12.84, *SE*_IG_ = 0.41; *p* = 0.053) and FU1 (*M*_CG.adj_ = 12.63, *SE*_CG_ = 0.50, *M*_*I*G.adj_ = 13.16, *SE*_IG_ = 0.47; *p* = 0.451). However, while the MoCA memory index score in the control group declined significantly over the three-month period (*p* = 0.008), the values in the intervention group remained, on average, stable (*p* = 0.496) (Figure 4A).

Looking at changes in subjective cognitive complaints, both groups did not differ at baseline (*M*_CG.adj_ = 6.07, *SE*_CG_ = 0.46, *M*_*I*G.adj_ = 6.22, *SE*_IG_ = 0.44; *p* = 0.811) or at FU1 (*M*_CG.adj_ = 5.54, *SE*_CG_ = 0.69, *M*_*I*G.adj_ = 3.69, *SE*_IG_ = 0.65; *p* = 0.065). Nevertheless, while subjective cognitive complaints in the control group remained unchanged on average (*p* = 0.387), a significant improvement was observed in individuals assigned to the intervention group (*p* < 0.001) (Figure 4B).

Finally, we found no group differences in depressive symptoms between the groups, neither at baseline (*M*_CG.adj_ = 16.02, *SE*_CG_ = 2.33, *M*_*I*G.adj_ = 18.59, *SE*_IG_ = 2.19; *p* = 0.431) nor FU1 (*M*_CG.adj_ = 14.34, *SE*_CG_ = 2.40, *M*_*I*G.adj_ = 11.81, *SE*_IG_ = 2.26; *p* = 0.452). However, while the intervention group was able to reduce their depressive symptoms from baseline to FU1 (*p* < 0.001), the depressive symptoms for the control group, on average, remained unchanged between the assessment time points (*p* = 0.328) (Figure 4C).

After all individuals who completed the training were merged to form a combined intervention group (*n* = 31), exploratory pre-post comparisons were conducted (pre values for intervention group: baseline; pre values for control group: follow-up 2; post values for intervention group: follow-up 1; post values for control group: follow-up 3), and the results are presented in Table 3.

**TABLE 3:** Results of the pre-post comparisons in all patients before and after completing the three-month tablet-based training.

Domain	Subtest	*t*	df	*p*	*d*	*M* ± *SD* (BL)	*M* ± *SD* (FU)
Attention	Digit span forward	−3.22	30	**0.003**	−0.58	7.19 ± 1.66	8.26 ± 1.86
	Digit span backward	−3.00	30	**0.005**	−0.54	5.06 ± 1.93	6.26 ± 2.34
	TMT-A [time]	3.56	30	**0.001**	0.64	31.75 ± 10.55	26.15 ± 10.26
Executive function	Planning	−1.22	30	0.230	−0.22	8.42 ± 2.64	9.13 ± 2.16
	Categories	−6.64	30	**<0.001**	−1.19	30.00 ± 8.99	38.71 ± 9.92
	TMT-B [time]	4.07	30	**<0.001**	0.73	63.25 ± 18.12	52.71 ± 17.20
Memory	Immediate recall (A)	1.23	30	0.229	0.22	29.69 ± 4.21	28.90 ± 4.42
	Immediate recall (B)	−2.98	30	**0.006**	−0.54	6.03 ± 2.04	7.42 ± 2.05
	Short-delayed recall	1.25	30	0.057	0.36	10.52 ± 1.96	9.90 ± 2.14
	Long-delayed recall	0.68	30	0.502	0.12	10.42 ± 2.26	10.16 ± 2.15
Word fluency	Formal-lexical	1.16	30	0.257	0.21	22.13 ± 1.22	20.13 ± 1.43
	Semantic	−1.56	30	0.130	−0.28	30.26 ± 11.26	34.45 ± 7.69
Global cognition	MoCA	−1.48	30	0.150	−0.27	28.26 ± 1.55	28.74 ± 1.24
	MoCA memory index	−1.68	30	0.103	−0.30	12.97 ± 1.74	13.58 ± 1.88
SCC	FSKB [QPC]	4.61	30	**<0.001**	0.83	5.42 ± 2.23	3.55 ± 2.34
Fatigue	FISD total	2.61	27	**0.015**	0.49	67.96 ± 29.40	56.29 ± 31.98
Depression	ADSL	7.69	30	**0.001**	0.63	16.81 ± 9.90	11.94 ± 8.91
	HADS-D	2.39	30	**0.024**	0.43	5.71 ± 4.74	4.19 ± 3.35
Anxiety	HADS-A	2.47	30	**0.020**	0.44	4.90 ± 3.28	4.00 ± 2.92
Quality of life	WHOQoL-Physical	−2.94	30	**0.006**	−0.53	24.84 ± 5.51	26.84 ± 5.44
	WHOQoL-Psychological	−2.77	30	**0.010**	−0.50	22.65 ± 3.56	23.71 ± 3.84

SCC, Subjective cognitive complaints. Significant results (pre-post changes) are highlighted in bold.

We found significant improvements in the digit span forward and digit span backward tasks, the TMT-A and TMT-B tasks, the categories task, the immediate recall of list B, subjective cognitive complaints, fatigue, depression, anxiety and quality of life (physical and psychological domain) after the training compared to before the training (Table 3). However, as discussed later, it cannot be ruled out that significant pre-post changes may have also occurred due to training effects (increased familiarity with the test material) or temporal improvement (symptom improvement over time on its own). Therefore, we performed a plausibility check and examined the course of the mean values of the original control group in all significant domains (from baseline to follow-up 2) in order to check if training effects or temporal improvement occurred without a training provided. Friedman tests were used to assess whether there were changes in the respective domains and subtests that occurred in a group without training (e.g., training/familiarity effects, or improvements over time in the control group).

After doing so, we can confirm reliable effects for the subtests Digit Span Backward (attention; *p* = 0.178), TMT-A (attention; *p* = 0.607), TMT-B (executive functions; *p* = 0.135), immediate recall wordlist B (memory; *p* = 0.390), FSKB [QPC] (subjective cognitive complaints; *p* = 0.083), FISD (fatigue; *p* = 0.052), ADSL (depression; *p* = 0.635), HADS-A (anxiety; *p* = 0.174) and WHOQoL quality of life (physical: *p* = 0.383, psychological: *p* = 0.794). As can be seen from the *p*-values, there were only negligible and non-significant changes between the time points (BL-FU1-FU2) in these domains in the original control group, suggesting that significant results from the paired *t*-tests in these domains (Table 3) can likely be attributed to a real intervention-related effect. As familiarity effects can never be ruled out with certainty, these results should be interpreted with caution, as discussed later. In addition, significant changes in the original control group, even without a training, where observed in the other domains (digit span forward: *p* = 0.030, categories: *p* = 0.002, HADS-D depression: *p* = 0.018).

### Association between symptom count and cognition

Furthermore, we were interested in exploring the correlation between the number of acute symptoms (symptoms reported during the initial Covid-19 illness) and cognition. We also investigated the same relationship for the number of persistent symptoms (post-Covid symptoms) reported by patients at baseline. The number of symptoms experienced during the initial Covid-19 infection was significantly positively correlated with the time needed to complete the NAB planning task (*r* = 0.43, *p* = 0.010), and negatively correlated with the immediate recall (A) score (*r* = −0.34, *p* = 0.038), the short-delayed recall (A) score (*r* = −0.34, *p* = 0.037), and the MoCA total score (*r* = −0.40, *p* = 0.015), while controlling for age, sex, and education.

**FIGURE 5:**
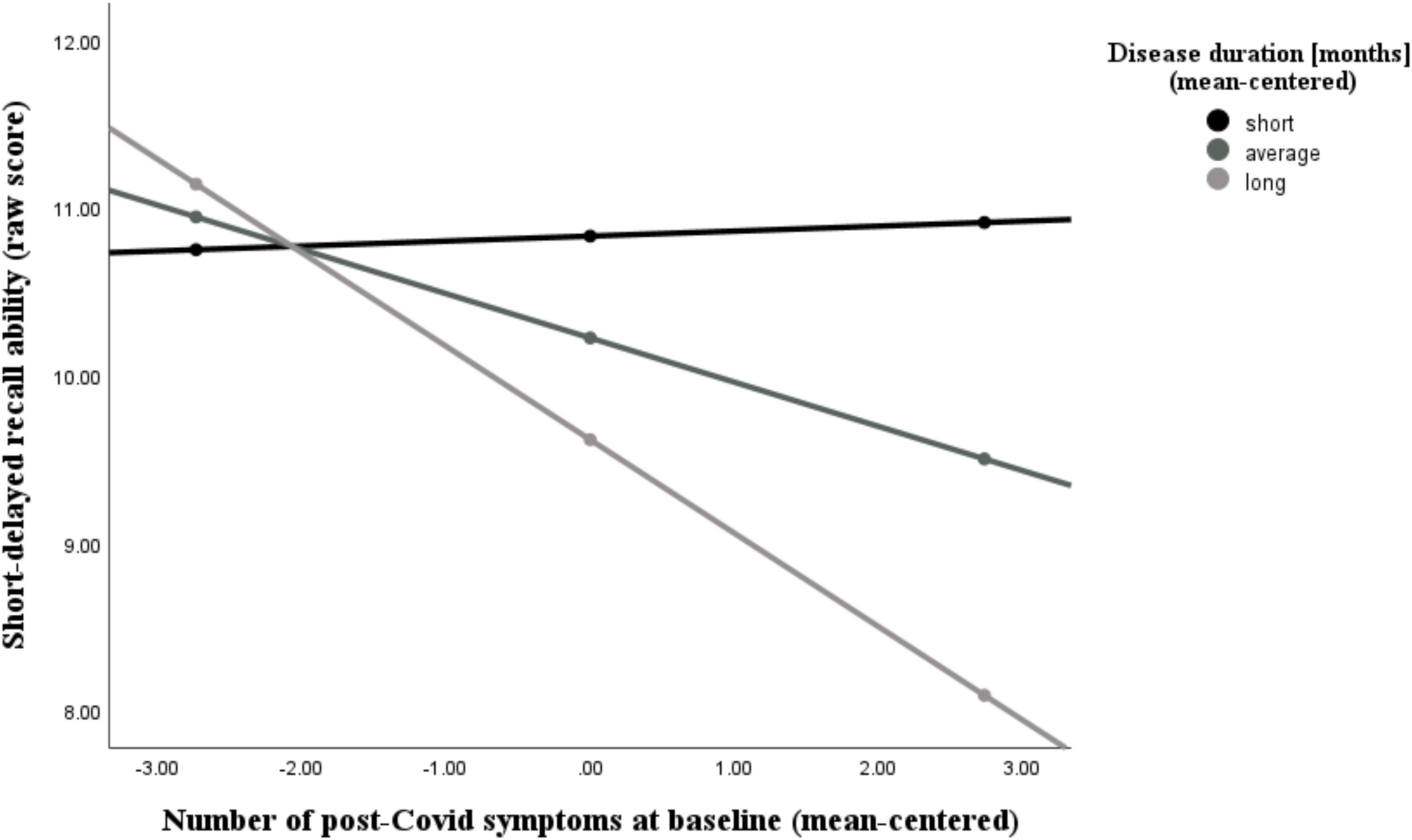
Moderating effect of post-Covid-19 disease duration on the association between the number of reported post-Covid symptoms and short-delayed recall ability. All variables that define the product term (i.e., predictor, moderator) were mean-centered. For individuals with a disease duration (months) one standard deviation below the mean (*M* = 18.26, *SD* = 10.31) in this sample (–1 *SD* below the mean; 7.95 months), no significant association between the number of post-Covid symptoms (BL) and their short-delayed recall ability was observed (*b* = 0.03, *SE*(HC3) = 0.16, *t* = 0.18, *p* = *0.855*). However, for those with an average disease duration, there was a significant negative correlation between symptom count and their short-term memory (*b* = –0.26, *SE*(HC3) = 0.10, *t* = –2.66, *p* = *0.012*). This negative effect is further intensified in individuals with a longer disease duration (+ 1 *SD* above the mean; 28.57 months) (*b* = –0.56, *SE*(HC3) = 0.08, *t* = –6.74, *p* < 0.001). Analyses are adjusted for age, sex, and education.

**FIGURE 6:**
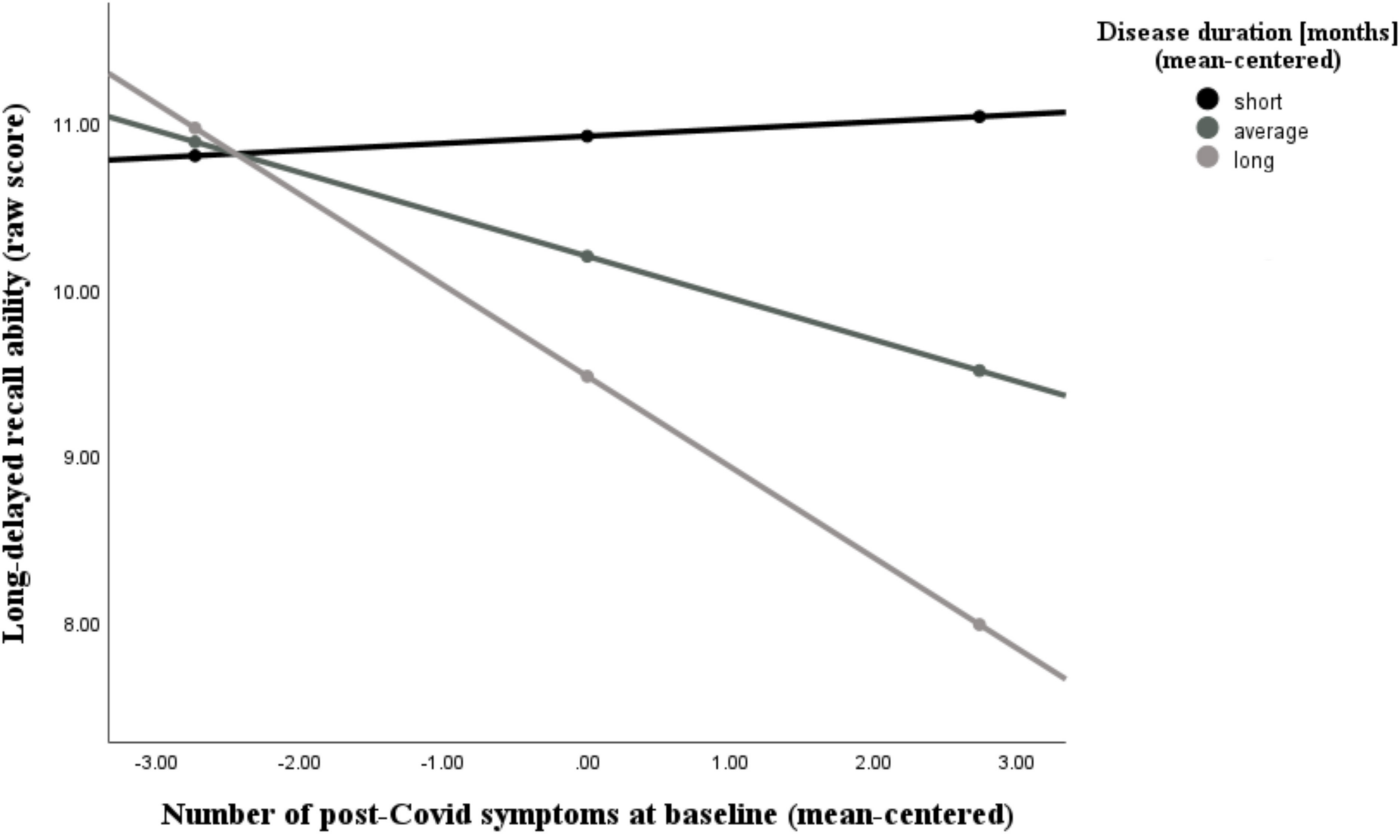
Moderating effect of post-Covid-19 disease duration on the association between the number of reported post-Covid symptoms and long-delayed recall ability. All variables that define the product term (i.e., predictor, moderator) were mean-centered. For individuals with a disease duration (months) one standard deviation below the mean (*M* = 18.26, *SD* = 10.31) in this sample (–1 *SD* below the mean; 7.95 months), no significant association between the number of post-Covid symptoms (BL) and their long-delayed recall ability was observed (*b* = 0.04, *SE*(HC3) = 0.18, *t* = 0.24, *p* = 0.812). Also, for those with an average disease duration, there was a visible, but non-significant correlation between symptom count and long-term memory (*b* = –0.25, *SE*(HC3) = 0.12, *t* = –2.04, *p* = 0.050). This negative effect is further intensified and statistically significant in individuals with a longer disease duration (+ 1 *SD* above the mean; 28.57 months) (*b* = –0.55, *SE*(HC3) = 0.13, *t* = –4.35, *p* < 0.001). Analyses are adjusted for age, sex, and education.

Additionally, we found that the number of post-Covid symptoms at baseline was significantly associated with the NAB digit span forward task (*r* = −0.44, *p* = 0.006), the immediate recall (A) score (*r* = −0.46, *p* = 0.004), the short-delayed recall (A) score (*r* = −0.46, *p* = 0.005), and the long-delayed recall (A) score (*r* = −0.39, *p* = 0.017), while controlling for age, sex, and education. The latter two abilities (short- and long-delayed recall) were further moderated by participants’ disease duration, as can be seen in Figures 5, 6 and Tables 4, 5. With increases in disease duration, the negative association between the number of post-Covid symptoms and patients’ short delayed-recall ability increases (gets even more negative), which indicates that a longer disease duration worsens the impact of post-Covid symptoms on memory. Equivalently, this applies to the long-delayed recall ability.

**TABLE 4:** Moderating effect of post-Covid-19 disease duration on the association between post-Covid symptom count and short-delayed recall ability in patients suffering from post-Covid-19 condition.

	Coefficient (*b*)	SE (HC3)	*t*	*p*	LLCI	ULCI
Constant	8.09	2.43	3.33	0.002	3.15	13.02
Ongoing symptom count	−0.26	0.10	−2.66	0.012	−0.47	−0.06
Disease duration [months]	−0.06	0.03	−1.93	0.062	−0.12	0.003
Interaction [P × M]	−0.03	0.01	−3.62	0.001	−0.04	−0.01

To enhance the interpretability of main effects, all terms that define the interaction (i.e., predictor [P] and moderator [M]) were mean centered. Analyses are adjusted for age, sex, and education. In addition, robust standard errors of type HC3 (Davidson-MacKinnon) were used. Model: *R*^2^ = 0.47, *F*(6, 33) = 12.76, *p* < 0.001; Interaction (symptoms x disease duration): *b* = −0.03 (*SE* = 0.01), *t* = −3.62, *p* = 0.001.

**TABLE 5:** Moderating effect of post-Covid-19 disease duration on the association between post-Covid symptom count and long-delayed recall ability in patients suffering from post-Covid-19 condition.

	Coefficient (*b*)	SE (HC3)	*t*	*p*	LLCI	ULCI
Constant	6.66	3.21	2.08	0.046	0.13	13.19
Ongoing symptom count	−0.25	0.12	−2.04	0.050	−0.50	−0.001
Disease duration [months]	−0.07	0.03	−2.11	0.042	−0.14	−0.003
Interaction [P × M]	−0.03	0.01	−3.16	0.003	−0.05	−0.01

To enhance the interpretability of main effects, all terms that define the interaction (i.e., predictor [P] and moderator [M]) were mean centered. Analyses are adjusted for age, sex, and education. In addition, robust standard errors of type HC3 (Davidson-MacKinnon) were used. Model: *R*^2^ = 0.41, *F*(6, 33) = 5.28, *p* < 0.001; Interaction (symptoms x disease duration): *b* = −0.03 (*SE* = 0.01), *t* = −3.16, *p* = 0.003.

There was no significant association between disease duration and post-Covid symptom count (*r* = −0.12, *p* = 0.486), as well as between disease duration and cognition (all *p-values* > 0.05), after controlling for age, sex, and education.

Finally, subjective cognitive complaints (FSKB [QPC] score) at baseline were not correlated with any cognitive or psychological domain assessed in this study (all *p*-values > 0.05; correlations with cognitive variables were adjusted for age, sex, and education).

## Discussion

Post-Covid-19 condition (PCC) represents a debilitating illness for affected individuals, characterized by a variety of physical, psychological, and cognitive symptoms (Li et al., 2023; Bonfim et al., 2024; Möller et al., 2023). However, there is still no gold standard for treating these symptoms (Mueller et al., 2023), and only a few studies have tested the efficacy of tailored and multidisciplinary training programs. To overcome the lack of interventional studies and to follow the advice of a previous review, suggesting digital interventions for patients with PCC to better manage their symptoms (Rinn et al., 2023), this study investigated (1) the efficacy of a three-month tablet-based training program, aiming to improve cognitive and mental symptoms, (2) the frequency of persistent symptoms of Covid-19 as well as the incidence of cognitive and mental deficits, and (3) the association between post-Covid symptoms and disease duration with cognition.

### Efficacy of a three-month tablet-based training program

Our findings revealed significant improvements in the intervention group compared to the control group regarding subjective cognitive complaints and depression. Furthermore, we observed that the MoCA Memory Index Score (MIS) did not deteriorate in the intervention as compared to the control group, suggesting a potential preservation of cognitive functions. These results suggest that the training had positive effects on the mental and emotional well-being of our patients. This aligns with positive effects of previous training studies in other populations, such as patients with Parkinson’s disease (Gavelin et al., 2022), or stroke (Zhou et al., 2022). We believe that the combination of relaxation exercises, which are known to be beneficial to improve mental health (e.g., depression) (Jia et al., 2020) and the cognitive training led to a decrease in subjective cognitive complaints of patients, which subsequently might have resulted in a decrease of depressive symptoms. As a result of cognitive training (especially memory exercises of the training), patients in the intervention group, as compared to those in the wait-list control group, showed no decline in the MoCA memory index score. As we know from previous literature that symptoms are fluctuating over time (Ziauddeen et al., 2022), being part of the intervention group might have counteracted a fluctuation in memory performance. However, in the relatively small sample of this study, our training did not yield significant improvements in other domains such as attention or executive functions, suggesting that the training may have limitations in targeting these specific cognitive domains or that the intervention duration was insufficient to yield measurable changes. This might have been due to small, but possibly meaningful effects which were not detectable. Additionally, the training intensity (i.e., the number of session participants completed per week) might have been insufficient. Another reason might be task specificity, meaning that the cognitive exercises may have focused more on memory and subjective complaints and less on executive functioning and other domains. Moreover, the absence of long-term effects indicates the necessity for more intensified interventions to achieve sustainable effects. Therefore, future research should explore strategies to prolong the effects of such interventions. Additionally, the training program needs to be adjusted in order to also induce improvements in other domains (e.g., executive functions, attention, or fatigue). For example, planning tasks could be incorporated, in which patients plan a daily schedule or a shopping list, or tasks that specifically target the subdomains of attention (e.g., divided attention, selective attention, sustained attention). Notably, we also found no effects with respect to fatigue, although fatigue represents one of the most common symptoms in PCC (Leitner et al., 2024). This negative finding highlights the need for future studies to develop interventions targeting fatigue. In addition, the impact of significant training effects on patients’ daily functionality could be further assessed through quantitative (e.g., questionnaires on activities of daily living) and qualitative interviews (e.g., focus groups) in future studies, which provide an extension to quantitative research findings. Finally, no negative side-effects were reported by patients.

Although the training had a significant effect on the domains mentioned above, it is further essential to note that participants in the intervention group either showed a decline in performance again after stopping the training (FU1 - FU2), or the control group improved from FU1 to FU2 even without training. This would suggest that effects achieved through the training are not stable and further, longer training periods are needed, or that training leads to quicker improvement in specific domains, but these improvements also occur spontaneously over time. A spontaneous improvement of post-Covid symptoms over time was also reported by previous studies (Derksen et al., 2023; Jason et al., 2021; Oliveira et al., 2022), although not necessarily for neurocognitive symptoms (Jason et al., 2021). Tröscher et al. (2024), for instance, reported that around three-quarters of patients showed an improvement of symptoms after approximately 18 months (Tröscher et al., 2024). This gives hope to patients currently suffering from lingering Covid-19 symptoms. Nonetheless, as persistent symptoms following a SARS-CoV-2 infection can be significantly debilitating and lead to limitations in the work and private life of those affected (Leitner et al., 2024; Walker et al., 2023; Ziauddeen et al., 2022), training options that lead to a faster improvement of symptoms are essential, especially since not all patients seem to recover spontaneously. A faster improvement of symptoms might help individuals in times of uncertainty regain their quality of life, reduce the risk of anxiety and depression, and allow them to return to work earlier.

After all individuals, including those in the waitlist control group, completed the tablet training, we merged both groups to form a combined intervention group. This allowed us to increase statistical power (the likelihood of detecting even small effects if they exist) by enlarging the sample size for further exploratory analyses. We found significant improvements in attention (3/3 tests), executive functions (2/3 tests), memory (1/4 tests), subjective cognitive complaints (1/1 test), fatigue (1/1 test), depression (2/2 tests), anxiety (1/1 test), and quality of life (2/2 tests). Although these results are remarkable, we cannot definitively rule out a practice/training effect. Therefore, we subsequently examined only those domains in which the original control group (before combining the groups) did not show any improvements during the period in which they did not receive training (BL - FU2). We found a significant improvement in some tests of the aforementioned domains, as the initial control group (before combining the groups) did not show any improvements (indicating that practice/familiarity effects or spontaneous improvements are unlikely). However, it is important to note that these findings are preliminary and should be validated in larger studies to definitively rule out familiarity effects or spontaneous improvement of symptoms over time. Additionally, seasonal variations and effects such as regression to the mean could not be controlled in this scenario.

As cognitive deficits might even persist for more than 2 years post-infection (Cheetham et al., 2023), and roughly 1 in 5 might not be able to work at all due to their symptoms (Walker et al., 2023), effective tabled-based interventions need to be incorporated into the treatment plan of patients with PCC (Derksen et al., 2023). Trainings should take specific care for patients that are suffering from severe fatigue, as physical or psychological exhaustion is reached easily, which might lead to negative consequences. In addition, future studies should investigate cognitive and mental training in larger samples in order to substantiate the effectiveness of such programs and address the issue of how long training sessions should last to achieve sustainable effects.

### Frequency of cognitive and mental deficits

In our study, individuals suffering from post-Covid reported a variety of different symptoms, including cognition-related symptoms such as memory problems (85.0%) or brain fog (87.5%), as well as symptoms such as fatigue (40.0%) and muscle pain (27.5%). At baseline, almost all individuals reported subjective cognitive complaints (97.5%). However, this might be attributed to a sampling bias, as recruitment for the study specifically focused on individuals with cognitive and mental deficits in the context of post-Covid. In addition, although we asked patients explicitly to provide only symptoms that have not occurred already prior to the illness, we cannot rule out that some symptoms occurred due to cognitive decline which is commonly observed in the aging population. Furthermore, we found attention deficits in 17.5% of all participants, memory problems in 12.5%, as well as a high rate of psychological symptoms including depression (up to 30.0%), anxiety (22.5%), or reduced quality of life across various domains (up to 36.1%). These results are in line with a number of previously published studies, indicating a high amount of cognitive (Cheetham et al., 2023; Lauria et al., 2023), psychological (Goodman et al., 2023) and physical symptoms (Byambasuren et al., 2023) in PCC. Although the number of patients with subjective cognitive complaints was substantial, these perceived deficits were interestingly not correlated with cognition as assessed during comprehensive neuropsychological testing. Findings like these are not unusual, as previous research indicated that subjective cognitive complaints are not necessarily associated with actual cognitive deficits in different populations such as those with bipolar disorders (Svendsen et al., 2012) or traumatic brain injury (French et al., 2014). Also, in a recently published study on patients with PCC, a high number of patients reported memory and concentration problems but showed no difference in cognition compared to healthy controls (Chang et al., 2023). This might suggest that some individuals may have difficulties in accurately assessing their cognitive abilities, although these complaints are representing a significant burden for those affected. Finally, and in line with Hasting et al. (2023), although there was a high prevalence of deficits in specific domains, severe cognitive impairment was relatively rare in our study sample. The absent of severe cognitive impairment was particularly visible when focusing on global screening instruments such as the MoCA (Hasting et al., 2023).

### Association between acute/post-Covid symptoms and disease duration with cognition

Finally, we could validate that PCC is associated with objectively measurable cognitive deficits (Cheetham et al., 2023; Hampshire et al., 2024; Houben and Bonnechère, 2022). The number of symptoms experienced during a patients’ acute Covid-19 illness was negatively associated with the performance in tests assessing executive functions, memory, and general cognition (MoCA) at the baseline assessment. This result is significant, as it illustrates that the number of acute symptoms correlates with cognitive performance in these domains, even though the acute infection occurred months ago. A similar result emerged when focusing on persistent (post-Covid) symptoms. The number of post-Covid symptoms at baseline was associated with a decrease in attention and memory abilities. The relationship between the amount of post-Covid symptoms with short and long-delayed recall abilities was further amplified by patients’ disease duration. For individuals with a relatively short duration of post-Covid illness, there was no correlation between symptom count and memory performance (i.e., regardless of how many or few symptoms were present, there was no effect on performance in memory tests). In contrast, for individuals with a relatively long duration of post-Covid illness, a higher number of post-Covid symptoms led to poorer short- and long-term memory performance. These results are a first indicator which suggests that training may be most beneficial when offered early in the course of the illness, as the impact of symptoms appears to become more prominent over time. However, it is important to note that further research is needed to confirm the optimal timing for long-Covid interventions.

In addition, post-Covid disease duration was not associated with post-Covid symptom count. This contrasts previous studies, suggesting that most symptoms show a decreasing prevalence over time (i.e., with increasing disease duration) (Derksen et al., 2023; Tran et al., 2022). However, it is likely that symptoms are fluctuating or relapsing over time (Ziauddeen et al., 2022) and that the prevalence (increase or decrease of symptoms over time) depends on the type of symptoms (Tran et al., 2022).

### Limitations

Despite numerous strengths of the study, such as comprehensive neuropsychological testing and the longitudinal study design, it is important to note some limitations as well. The impact of tablet-based interventions may mainly consist of smaller effects, which can only be detected in larger samples (hence requiring greater statistical power). The relatively small sample size per group [as the study design focused on medium to large effects (see power analysis)] could account for some non-significant effects that might be detected in studies with a larger sample size. Future studies should consider using more conservative effect size assumptions and recruiting larger samples to improve the generalizability of their findings. We also suggest an exact recording of how often and for how long participants perform the exercises to obtain a clearer picture of retention and adherence. Another point to consider is that while we found more effects after merging both groups into a single intervention group, we cannot rule out the possibility that these effects arose due to familiarity with the test material or spontaneous remission over time. Also, seasonal variations and phenomena such as regression to the mean could not be controlled in these exploratory analyses. These results should therefore not be overinterpreted. Therefore, future studies should focus on a similar training design with a larger sample size to examine smaller but meaningful effects that can be achieved through tablet-based interventions. In this regard, we recommend that future studies also consider differences in depressive symptoms between groups (as they can affect cognition) and include an active control group in their study design. This active control group should receive an alternative intervention that is comparable in effort and duration but does not include the active component of the main intervention (e.g., exercises to improve cognitive functioning). This approach could significantly improve the generalizability of the results. For recruitment, we recommend ensuring that all age groups, races, and genders have equal opportunities to participate in future studies. Another general limitation of tablet-based interventions, as conducted in this study, is the fact that the patients could not be blinded to the treatment and therefore knew whether they belonged to the intervention or control group. We also want to acknowledge that this study was only registered retrospectively and not prospectively, which we encourage future studies to do. A final limitation of the study concerns the transferability of the effects into the daily life, as it remains unknown whether the training also improved participants’ everyday functioning.

## Conclusion

Our study suggests that home- and tablet-based cognitive and mental training may be partially effective in improving specific symptoms associated with Post-Covid-19 condition (PCC). Participants in the intervention group showed a significant reduction in subjective cognitive complaints and depressive symptoms compared to a control group. Additionally, their MoCA Memory Index Score remained stable, while it declined significantly over time in the wait-list control group. From a clinical perspective, such interventions could provide a valuable and flexible option to support cognitive and psychological recovery in patients suffering from PCC. Finally, we found preliminary evidence that the negative association between the number of post-Covid symptoms and participants’ memory performance becomes more pronounced with increased disease duration. This is a first indicator which suggests that training should be provided early in the course of the illness. Future studies with larger sample sizes should include an active control group and focus more on the transferability of effects into patients’ daily lives. Overall, our findings suggest that tablet-based training programs could be considered an additional add-on therapy to improve cognition and mental health in patients suffering from post-Covid-19 symptoms.

## Data Availability

The raw data supporting the conclusions of this article will be made available by the authors, without undue reservation.

## Footnotes

1
www.processmacro.org

## References

[B1] Abdelrahman M. M. Abd-Elrahman N. M. Bakheet T. M. (2021). Persistence of symptoms after improvement of acute COVID19 infection, a longitudinal study. *J. Med. Virol.* 93 5942–5946. 10.1002/jmv.27156 34171139 PMC8426945

[B2] Altmann D. M. Whettlock E. M. Liu S. Arachchillage D. J. Boyton R. J. (2023). The immunology of long COVID. *Nat. Rev. Immunol.* 23 618–634. 10.1038/s41577-023-00904-7 37433988

[B3] Angermeyer M. C. Kilian R. Matschinger H. (2000). *WHOQOL-100 und WHOQOL-BREF: Handbuch für die deutschsprachigen versionen der WHO instrumente zur erfassung von lebensqualität. [WHOQOL-100 and WHOQOL-BREF: Manual for the German versions of the WHO instruments for assessing quality of life].* Gottingen: Hogrefe. German.

[B4] Arbuthnott K. Frank J. (2000). Trail making test, part B as a measure of executive control: Validation using a set-switching paradigm. *J. Clin. Exp. Neuropsychol.* 22 518–528. 10.1076/1380-3395(200008)22:4;1-0;FT518 10923061

[B5] Aschenbrenner S. Tucha O. Lange K. W. (2001). *Regensburger wortflüssigkeits-test. [Regensburg word fluency test].* Göttingen: Hogrefe, Verlag für Psychologie. German.

[B6] Bahar-Fuchs A. Barendse M. E. A. Bloom R. Ravona-Springer R. Heymann A. Dabush H. (2020). Computerized cognitive training for older adults at higher dementia risk due to diabetes: Findings from a randomized controlled trial. *J. Gerontol. A Biol. Sci. Med. Sci.* 75 747–754. 10.1093/gerona/glz073 30868154 PMC7931965

[B7] Ballering A. V. van Zon S. K. R. Olde Hartman T. C. Rosmalen J. G. M. (2022). Persistence of somatic symptoms after COVID-19 in the Netherlands: An observational cohort study. *Lancet* 400 452–461. 10.1016/S0140-6736(22)01214-4 35934007 PMC9352274

[B8] Barthélémy H. Mougenot E. Duracinsky M. Salmon-Ceron D. Bonini J. Péretz F. (2022). Smoking increases the risk of post-acute COVID-19 syndrome: Results from a French community-based survey. *Tob. Induc. Dis.* 20:59. 10.18332/tid/150295 35799625 PMC9204712

[B9] Bonfim L. P. F. Correa T. R. Freire B. C. C. Pedroso T. M. Pereira D. N. Fernandes T. B. (2024). Post-COVID-19 cognitive symptoms in patients assisted by a teleassistance service: A retrospective cohort study. *Front. Public Health* 12:1282067. 10.3389/fpubh.2024.1282067 38689777 PMC11060150

[B10] Boufidou F. Medić S. Lampropoulou V. Siafakas N. Tsakris A. Anastassopoulou C. (2023). SARS-CoV-2 reinfections and long COVID in the post-omicron phase of the pandemic. *Int. J. Mol. Sci.* 24:12962. 10.3390/ijms241612962 37629143 PMC10454552

[B11] Byambasuren O. Stehlik P. Clark J. Alcorn K. Glasziou P. (2023). Effect of COVID-19 vaccination on long COVID: Systematic review. *BMJ Med.* 2:e000385. 10.1136/bmjmed-2022-000385 36936268 PMC9978692

[B12] Carfi A. Bernabei R. Landi F. Gemelli Against COVID-19 Post-Acute Care Study Group. (2020). Persistent symptoms in patients after acute COVID-19. *JAMA* 324 603–605. 10.1001/jama.2020.12603 32644129 PMC7349096

[B13] Ceban F. Ling S. Lui L. M. W. Lee Y. Gill H. Teopiz K. M. (2022). Fatigue and cognitive impairment in Post-COVID-19 syndrome: A systematic review and meta-analysis. *Brain Behav. Immun.* 101 93–135. 10.1016/j.bbi.2021.12.020 34973396 PMC8715665

[B14] Chang L. Ryan M. C. Liang H. Zhang X. Cunningham E. Wang J. (2023). Changes in brain activation patterns during working memory tasks in people with post-COVID condition and persistent neuropsychiatric symptoms. *Neurology* 100 e2409–e2423. 10.1212/WNL.0000000000207309 37185175 PMC10256123

[B15] Cheetham N. J. Penfold R. Giunchiglia V. Bowyer V. Sudre C. H. Canas L. S. (2023). The effects of COVID-19 on cognitive performance in a community-based cohort: A COVID symptom study biobank prospective cohort study. *EClinicalMedicine* 62:102086. 10.1016/j.eclinm.2023.102086 37654669 PMC10466229

[B16] Cipolli G. C. Alonso V. Yasuda C. L. Assumpção D. Cachioni M. Melo R. C. (2023). Cognitive impairment in post-acute COVID-19 syndrome: A scoping review. *Arq. Neuropsiquiatr.* 81 1053–1069. 10.1055/s-0043-1777115 38157873 PMC10756850

[B17] Dacosta-Aguayo R. Puig J. Lamonja-Vicente N. Carmona-Cervelló M. León-Gómez B. B. Monté-Rubio G. (2024). Reduced cortical thickness correlates of cognitive dysfunction in post-COVID-19 condition: Insights from a long-term follow-up. *AJNR Am. J. Neuroradiol.* 45 647–654. 10.3174/ajnr.A8167 38575319 PMC11288549

[B18] Davis H. E. Mccorkell L. Vogel J. M. Topol E. J. (2023). Long COVID: Major findings, mechanisms and recommendations. *Nat. Rev. Microbiol.* 21:408. 10.1038/s41579-023-00896-0 37069455 PMC10408714

[B19] Derksen C. Rinn R. Gao L. Dahmen A. Cordes C. Kolb C. (2023). Longitudinal evaluation of an integrated post-COVID-19/Long COVID management program consisting of digital interventions and personal support: Randomized controlled trial. *J. Med. Intern. Res.* 25:e49342. 10.2196/49342 37792437 PMC10563866

[B20] Di Gennaro F. Belati A. Tulone O. Diella L. Fiore Bavaro D. Bonica R. (2023). Incidence of long COVID-19 in people with previous SARS-Cov2 infection: A systematic review and meta-analysis of 120,970 patients. *Intern. Emerg. Med.* 18 1573–1581. 10.1007/s11739-022-03164-w 36449260 PMC9709360

[B21] Duñabeitia J. A. Mera F. Baro Ó Jadad-Garcia T. Jadad A. R. (2023). Personalized computerized training for cognitive dysfunction after COVID-19: A before-and-after feasibility pilot study. *Int. J. Environ. Res. Public Health* 20:3100. 10.3390/ijerph20043100 36833793 PMC9966004

[B22] Faul F. Erdfelder E. Lang A. Buchner A. G. (2007). *Power 3: A flexible statistical power analysis program for the social, behavioral, and biomedical sciences. *Behav. Res. Methods* 39 175–191. 10.3758/bf03193146 17695343

[B23] Fernández-de-Las-Peñas C. Martín-Guerrero J. D. Pellicer-Valero ÓJ. Navarro-Pardo E. Gómez-Mayordomo V. Cuadrado M. L. (2022). Female sex is a risk factor associated with long-term post-COVID related-symptoms but not with COVID-19 symptoms: The Long-COVID-EXP-CM multicenter study. *J. Clin. Med.* 11:413. 10.3390/jcm11020413 35054108 PMC8778106

[B24] Fernández-de-Las-Peñas C. Palacios-Ceña D. Gómez-Mayordomo V. Florencio L. L. Cuadrado M. L. Plaza-Manzano G. (2021). Prevalence of post-COVID-19 symptoms in hospitalized and non-hospitalized COVID-19 survivors: A systematic review and meta-analysis. *Eur. J. Intern. Med.* 92 55–70. 10.1016/j.ejim.2021.06.009 34167876 PMC8206636

[B25] Fernández-de-Las-Peñas C. Raveendran A. V. Giordano R. Arendt-Nielsen L. (2023). Long COVID or Post-COVID-19 condition: Past, present and future research directions. *Microorganisms* 11:2959. 10.3390/microorganisms11122959 38138102 PMC10745830

[B26] Fleischer M. Szepanowski F. Tovar M. Herchert K. Dinse H. Schweda A. (2022). Post-COVID-19 syndrome is rarely associated with damage of the nervous system: Findings from a prospective observational cohort study in 171 patients. *Neurol. Ther.* 11 1637–1657. 10.1007/s40120-022-00395-z 36028604 PMC9417089

[B27] French L. M. Lange R. T. Brickell T. (2014). Subjective cognitive complaints and neuropsychological test performance following military-related traumatic brain injury. *J. Rehabil. Res. Dev.* 51 933–950. 10.1682/JRRD.2013.10.0226 25479042

[B28] Gavelin H. M. Domellöf M. E. Leung I. Neely A. S. Launder N. H. Nategh L. (2022). Computerized cognitive training in Parkinson’s disease: A systematic review and meta-analysis. *Ageing Res. Rev.* 80:101671. 10.1016/j.arr.2022.101671 35714854

[B29] Godoy-González M. Navarra-Ventura G. Gomà G. de Haro C. Espinal C. Fortià C. (2023). Objective and subjective cognition in survivors of COVID-19 one year after ICU discharge: The role of demographic, clinical, and emotional factors. *Crit. Care* 27:188. 10.1186/s13054-023-04478-7 37189173 PMC10184095

[B30] Gonzalez-Fernandez E. Huang J. (2023). Cognitive aspects of COVID-19. *Curr. Neurol. Neurosci. Rep.* 23 531–538. 10.1007/s11910-023-01286-y 37490194

[B31] Goodman M. L. Molldrem S. Elliott A. Robertson D. Keiser P. (2023). Long COVID and mental health correlates: A new chronic condition fits existing patterns. *Health Psychol. Behav. Med.* 11:2164498. 10.1080/21642850.2022.2164498 36643576 PMC9833408

[B32] Grant M. C. Geoghegan L. Arbyn M. Mohammed Z. McGuinness L. Clarke E. L. (2020). The prevalence of symptoms in 24,410 adults infected by the novel coronavirus (SARS-CoV-2; COVID-19): A systematic review and meta-analysis of 148 studies from 9 countries. *PLoS One* 15:e0234765. 10.1371/journal.pone.0234765 32574165 PMC7310678

[B33] Guillén N. Pérez-Millan A. Falgàs N. Lledó-Ibáñez G. M. Rami L. Sarto J. (2024). Cognitive profile, neuroimaging and fluid biomarkers in post-acute COVID-19 syndrome. *Sci. Rep.* 14:12927. 10.1038/s41598-024-63071-2 38839833 PMC11153491

[B34] Hagen B. I. Lerdal A. Søraas A. Landrø N. I. Bø R. Småstuen M. C. (2022). Cognitive rehabilitation in post-COVID-19 condition: A study protocol for a randomized controlled trial. *Contemp. Clin. Trials* 122:106955. 10.1016/j.cct.2022.106955 36208718 PMC9533592

[B35] Hampshire A. Azor A. Atchison C. Trender W. Hellyer P. J. Giunchiglia V. (2024). Cognition and memory after COVID-19 in a large community sample. *N. Engl. J. Med.* 390 806–818. 10.1056/NEJMoa2311330 38416429 PMC7615803

[B36] Han Q. Zheng B. Daines L. Sheikh A. (2022). Long-Term sequelae of COVID-19: A systematic review and meta-analysis of one-year follow-up studies on post-COVID symptoms. *Pathogens* 11:269. 10.3390/pathogens11020269 35215212 PMC8875269

[B37] Haslam A. Olivier T. Prasad V. (2023). The definition of long COVID used in interventional studies. *Eur. J. Clin. Invest.* 53:e13989. 10.1111/eci.13989 36964995

[B38] Hasting A. S. Herzig S. Obrig H. Schroeter M. L. Villringer A. Thöne-Otto A. I. T. (2023). The leipzig treatment program for interdisciplinary diagnosis and therapy of neurocognitive post-COVID symptoms. *Zeitschrift Neuropsychol.* 34 71–83. 10.1024/1016-264X/a000376

[B39] Häuser W. Almouhtasseb R. Muthny F. A. Grandt D. (2003). [Validation of a german version of the fatigue impact scale FIS-D]. *Z. Gastroenterol.* 41 973–982. 10.1055/s-2003-42927 14562194

[B40] Hautzinger M. Bailer M. Hofmeister D. Keller F. (2012). *Allgemeine depressionsskala, 2., überarbeitete und neu normierte auflage. [General depression scale, 2nd, revised and newly standardized edition].* Gottingen: Hogrefe. German.

[B41] Hayes A. F. (2022). *Introduction to mediation, moderation, and conditional process analysis: A regression-based approach.* New York, NY: The Guilford Press.

[B42] Hedberg P. Granath F. Bruchfeld J. Askling J. Sjöholm D. Fored M. (2023). Post COVID-19 condition diagnosis: A population-based cohort study of occurrence, associated factors, and healthcare use by severity of acute infection. *J. Intern. Med.* 293 246–258. 10.1111/joim.13584 36478477 PMC9877994

[B43] Herrera E. Pérez-Sánchez M. D. C. San Miguel-Abella R. Barrenechea A. Blanco C. Solares L. (2023). Cognitive impairment in young adults with post COVID-19 syndrome. *Sci. Rep.* 13:6378. 10.1038/s41598-023-32939-0 37076533 PMC10113715

[B44] Herrmann-Lingen C. Buss U. Snaith R. P. (2018). “Hospital anxiety and depression scale - deutsche version,” in *Deutsche adaptation der hospital anxiety and depression scale (HADS)*, eds von Snaith R. P. Zigmond A. S. (Gottingen: Hogrefe).

[B45] Hill N. T. Mowszowski L. Naismith S. L. Chadwick V. L. Valenzuela M. Lampit A. (2017). Computerized cognitive training in older adults with mild cognitive impairment or dementia: A systematic review and meta-analysis. *Am. J. Psychiatry* 174 329–340. 10.1176/appi.ajp.2016.16030360 27838936

[B46] Houben S. Bonnechère B. (2022). The impact of COVID-19 infection on cognitive function and the implication for rehabilitation: A systematic review and meta-analysis. *Int. J. Environ. Res. Public Health* 19:7748. 10.3390/ijerph19137748 35805406 PMC9266128

[B47] Hu M. Wu X. Shu X. Hu H. Chen Q. Peng L. (2021). Effects of computerised cognitive training on cognitive impairment: A meta-analysis. *J. Neurol.* 268 1680–1688. 10.1007/s00415-019-09522-7 31650255

[B48] Jacobson E. (1938). *Progressive relaxation*, 2nd Edn. Chicago, IL: University of Chicago Press.

[B49] Jason L. A. Islam M. Conroy K. Cotler J. Torres C. Johnson M. (2021). COVID-19 symptoms over time: Comparing long-haulers to ME/CFS. *Fatigue* 9 59–68. 10.1080/21641846.2021.1922140 34484973 PMC8411893

[B50] Jaywant A. Gunning F. M. Oberlin L. E. Santillana M. Ognyanova K. Druckman J. N. (2024). Cognitive symptoms of Post-COVID-19 condition and daily functioning. *JAMA Netw. Open* 7:e2356098. 10.1001/jamanetworkopen.2023.56098 38353947 PMC10867690

[B51] Jebrini T. Thomas A. Sachenbacher S. Heimkes F. Karch S. Goerigk S. (2024). Effects of cognitive training and group psychotherapy on cognitive performance of post COVID-19 patients: An exploratory and non-randomized clinical trial. *Eur. Arch. Psychiatry Clin. Neurosci.* 274 1969–1982. 10.1007/s00406-024-01904-x 39356325 PMC11579059

[B52] Jennings G. Monaghan A. Xue F. Duggan E. Romero-Ortuno R. (2022). Comprehensive clinical characterisation of brain fog in adults reporting long COVID symptoms. *J. Clin. Med.* 11:3440. 10.3390/jcm11123440 35743516 PMC9224578

[B53] Jia Y. Wang X. Cheng Y. (2020). Relaxation therapy for depression: An updated meta-analysis. *J. Nerv. Ment. Dis.* 208 319–328. 10.1097/NMD.0000000000001121 32221187

[B54] Joli J. Buck P. Zipfel S. Stengel A. (2022). Post-COVID-19 fatigue: A systematic review. *Front. Psychiatry* 13:947973. 10.3389/fpsyt.2022.947973 36032234 PMC9403611

[B55] Kim Y. Bae S. Chang H. H. Kim S. W. (2023). Long COVID prevalence and impact on quality of life 2 years after acute COVID-19. *Sci. Rep.* 13:11207. 10.1038/s41598-023-36995-4 37433819 PMC10336045

[B56] Klok F. A. Boon G. J. A. M. Barco S. Endres M. Geelhoed J. J. M. Knauss S. (2020). The Post-COVID-19 functional status scale: A tool to measure functional status over time after COVID-19. *Eur. Respir. J.* 56:2001494. 10.1183/13993003.01494-2020 32398306 PMC7236834

[B57] Koczulla A. R. Ankermann T. Behrends U. Berlit P. Boing S. Brinkmann F. (2021). [S1 guideline post-COVID/Long-COVID]. *Pneumologie* 75 869–900. 10.1055/a-1551-9734 34474488

[B58] Kozik V. Reuken P. Utech I. Gramlich J. Stallmach Z. Demeyere N. (2023). Characterization of neurocognitive deficits in patients with post-COVID-19 syndrome: Persistence, patients’ complaints, and clinical predictors. *Front. Psychol.* 14:1233144. 10.3389/fpsyg.2023.1233144 37915528 PMC10616256

[B59] Krishna B. Wills M. Sithole N. (2023). Long COVID: What is known and what gaps need to be addressed. *Br. Med. Bull.* 147 6–19. 10.1093/bmb/ldad016 37434326 PMC10502447

[B60] Lanz-Luces J. R. Aceituno H. Quiroz-Bravo F. Rodríguez-Flores F. Osores-Espinoza M. Rigaud D. (2022). Long-lasting brain fog is related with severity clusters of symptoms in COVID-19 patients. *Rev. Med. Chil.* 150 1484–1492. 10.4067/S0034-98872022001101484 37358174

[B61] Lauria A. Carfì A. Benvenuto F. Bramato G. Ciciarello F. Rocchi S. (2023). Neuropsychological measures of post-COVID-19 cognitive status. *Front. Psychol.* 14:1136667. 10.3389/fpsyg.2023.1136667 37492442 PMC10363721

[B62] Leitner M. Pötz G. Berger M. Fellner M. Spat S. Koini M. (2024). Characteristics and burden of acute COVID-19 and long-COVID: Demographic, physical, mental health, and economic perspectives. *PLoS One* 19:e0297207. 10.1371/journal.pone.0297207 38252638 PMC10802963

[B63] Li Z. Zhang Z. Zhang Z. Wang Z. Li H. (2023). Cognitive impairment after long COVID-19: Current evidence and perspectives. *Front. Neurol.* 14:1239182. 10.3389/fneur.2023.1239182 37583958 PMC10423939

[B64] Lippi G. Sanchis-Gomar F. Henry B. M. (2023). COVID-19 and its long-term sequelae: What do we know in 2023? *Pol. Arch. Intern. Med.* 133:16402. 10.20452/pamw.16402 36626183

[B65] Markova H. Andel R. Stepankova H. Kopecek M. Nikolai T. Hort J. (2017). Subjective cognitive complaints in cognitively healthy older adults and their relationship to cognitive performance and depressive symptoms. *J. Alzheimers Dis.* 59 871–881. 10.3233/JAD-160970 28697555

[B66] Martin E. M. Srowig A. Utech I. Schrenk S. Kattlun F. Radscheidt M. (2024). Persistent cognitive slowing in post-COVID patients: Longitudinal study over 6 months. *J. Neurol.* 271 46–58. 10.1007/s00415-023-12069-3 37936010 PMC10769987

[B67] McLaughlin M. Cerexhe L. Macdonald E. Ingram J. Sanal-Hayes N. E. M. Meach R. (2023). A cross-sectional study of symptom prevalence, frequency, severity, and impact of long COVID in Scotland: Part I. *Am. J. Med.* 138 121–130. 10.1016/j.amjmed.2023.07.004 37481021

[B68] McWhirter L. Smyth H. Hoeritzauer I. Couturier A. Stone J. Carson A. J. (2023). What is brain fog? *J. Neurol. Neurosurg. Psychiatry* 94 321–325. 10.1136/jnnp-2022-329683 36600580

[B69] Miskowiak K. W. Johnsen S. Sattler S. M. Nielsen S. Kunalan K. Rungby J. (2021). Cognitive impairments four months after COVID-19 hospital discharge: Pattern, severity and association with illness variables. *Eur. Neuropsychopharmacol.* 46 39–48. 10.1016/j.euroneuro.2021.03.019 33823427 PMC8006192

[B70] Möller M. Borg K. Janson C. Lerm M. Normark J. Niward K. (2023). Cognitive dysfunction in post-COVID-19 condition: Mechanisms, management, and rehabilitation. *J. Intern. Med.* 294 563–581. 10.1111/joim.13720 37766515

[B71] Mueller M. R. Ganesh R. Hurt R. T. Beckman T. J. (2023). Post-COVID conditions. *Mayo Clin. Proc.* 98 1071–1078. 10.1016/j.mayocp.2023.04.007 37419575

[B72] Munblit D. O’Hara M. E. Akrami A. Perego E. Olliaro P. Needham D. M. (2022). Long COVID: Aiming for a consensus. *Lancet Respir. Med.* 10 632–634. 10.1016/S2213-2600(22)00135-7 35525253 PMC9067938

[B73] Nalbandian A. Desai A. D. Wan E. Y. (2023). Post-COVID-19 condition. *Annu. Rev. Med.* 74 55–64. 10.1146/annurev-med-043021-030635 35914765

[B74] Nasreddine Z. S. Phillips N. A. Bédirian V. Charbonneau S. Whitehead V. Collin I. (2005). The montreal cognitive assessment, MoCA: A Brief Screening Tool For Mild Cognitive Impairment. *J. Am. Geriatr. Soc.* 53 695–699. 10.1111/j.1532-5415.2005.53221.x 15817019

[B75] Nice (2020). *COVID-19 rapid guideline: Managing the long-term effects of COVID-19.* London: National Institute for Health and Care Excellence. 33555768

[B76] Nurek M. Rayner C. Freyer A. Taylor S. Järte L. MacDermott N. (2021). Recommendations for the recognition, diagnosis, and management of long COVID: A Delphi study. *Br. J. Gen. Pract.* 71 e815–e825. 10.3399/BJGP.2021.0265 34607799 PMC8510689

[B77] Oliveira C. R. Jason L. A. Unutmaz D. Bateman L. Vernon S. D. (2022). Improvement of long COVID symptoms over one year. *Front. Med.* 9:1065620. 10.3389/fmed.2022.1065620 36698810 PMC9868805

[B78] O’Mahoney L. L. Routen A. Gillies C. Ekezie W. Welford A. Zhang A. (2023). The prevalence and long-term health effects of long COVID among hospitalised and non-hospitalised populations: A systematic review and meta-analysis. *EClinicalMedicine* 55:101762. 10.1016/j.eclinm.2022.101762 36474804 PMC9714474

[B79] Palladini M. Bravi B. Colombo F. Caselani E. Di Pasquasio C. D’Orsi G. (2023). Cognitive remediation therapy for post-acute persistent cognitive deficits in COVID-19 survivors: A proof-of-concept study. *Neuropsychol. Rehabil.* 33 1207–1224. 10.1080/09602011.2022.2075016 35583357

[B80] Petermann F. Jäncke L. Waldmann H. C. (2013). *Neuropsychological assessment battery: Deutschsprachige adaptation der Neuropsychological Assessment Battery (NAB).* Gottingen: Hogrefe.

[B81] Reitan R. M. (1958). Validity of the trail making test as an indicator of organic brain damage. *Percep. Motor Skills* 8 271–276. 10.2466/pms.1958.8.3.271

[B82] Rinn R. Gao L. Schoeneich S. Dahmen A. Anand Kumar V. Becker P. (2023). Digital interventions for treating post-COVID or long-COVID symptoms: Scoping review. *J. Med. Intern. Res.* 25:e45711. 10.2196/45711 36943909 PMC10131666

[B83] Ruzicka M. Sachenbacher S. Heimkes F. Uebleis A. O. Karch S. Grosse-Wentrup F. (2024). Characterization of cognitive symptoms in post COVID-19 patients. *Eur. Arch. Psychiatry Clin. Neurosci.* 274 1923–1934. 10.1007/s00406-024-01821-z 38739263 PMC11579195

[B84] Scardua-Silva L. Amorim da Costa B. Karmann Aventurato Í Batista Joao R. Machado de Campos B. Rabelo de Brito M. (2024). Microstructural brain abnormalities, fatigue, and cognitive dysfunction after mild COVID-19. *Sci. Rep.* 14:1758. 10.1038/s41598-024-52005-7 38242927 PMC10798999

[B85] Seighali N. Abdollahi A. Shafiee A. Amini M. J. Teymouri Athar M. M. Safari O. (2024). The global prevalence of depression, anxiety, and sleep disorder among patients coping with Post COVID-19 syndrome (long COVID): A systematic review and meta-analysis. *BMC Psychiatry* 24:105. 10.1186/s12888-023-05481-6 38321404 PMC10848453

[B86] Subramanian A. Nirantharakumar K. Hughes S. Myles P. Williams T. Gokhale K. M. (2022). Symptoms and risk factors for long COVID in non-hospitalized adults. *Nat. Med.* 28 1706–1714. 10.1038/s41591-022-01909-w 35879616 PMC9388369

[B87] Sudre C. H. Murray B. Varsavsky T. Graham M. S. Penfold R. S. Bowyer R. C. (2021). Attributes and predictors of long COVID. *Nat. Med.* 27 626–631. 10.1038/s41591-021-01292-y 33692530 PMC7611399

[B88] Svendsen A. M. Kessing L. V. Munkholm K. Vinberg M. Miskowiak K. W. (2012). Is there an association between subjective and objective measures of cognitive function in patients with affective disorders? *Nord. J. Psychiatry* 66 248–253. 10.3109/08039488.2011.626870 22070515

[B89] Sykes D. L. Holdsworth L. Jawad N. Gunasekera P. Morice A. H. Crooks M. G. (2021). Post-COVID-19 symptom burden: What is long-COVID and how should we manage it? *Lung* 199 113–119. 10.1007/s00408-021-00423-z 33569660 PMC7875681

[B90] Tene L. Bergroth T. Eisenberg A. David S. S. B. Chodick G. (2023). Risk factors, health outcomes, healthcare services utilization, and direct medical costs of patients with long COVID. *Int. J. Infect. Dis.* 128 3–10. 10.1016/j.ijid.2022.12.002 36529373

[B91] Thams F. Antonenko D. Fleischmann R. Meinzer M. Grittner U. Schmidt S. (2022). Neuromodulation through brain stimulation-assisted cognitive training in patients with post-COVID-19 cognitive impairment (Neuromod-COV): Study protocol for a PROBE phase IIb trial. *BMJ Open* 12:e055038. 10.1136/bmjopen-2021-055038 35410927 PMC9002255

[B92] Thomas-Antérion C. Ribas C. Honore-Masson S. Million J. Laurent B. (2004). Evaluation de la plainte cognitive de patients Alzheimer, de sujets MCI, anxiodépressifs et de témoins avec le QPC (Questionnaire de Plainte Cognitive). [Assessment of cognitive complaints in Alzheimer’s patients, MCI subjects, anxiety-depressives and controls with the QPC (Cognitive Complaint Questionnaire)]. *NPG Neurol. Psychiatrie Gériatrie* 4 30–34. 10.1016/S1627-4830(04)97931-7 French

[B93] Thompson E. J. Williams D. M. Walker A. J. Mitchell R. E. Niedzwiedz C. L. Yang T. C. (2022). Long COVID burden and risk factors in 10 UK longitudinal studies and electronic health records. *Nat. Commun.* 13:3528. 10.1038/s41467-022-30836-0 35764621 PMC9240035

[B94] Torrell G. Puente D. Jacques-Aviñó C. Carrasco-Ribelles L. A. Violán C. López-Jiménez T. (2024). Characterisation, symptom pattern and symptom clusters from a retrospective cohort of Long COVID patients in primary care in Catalonia. *BMC Infect. Dis.* 24:82. 10.1186/s12879-023-08954-x 38225587 PMC10789045

[B95] Tran V. T. Porcher R. Pane I. Ravaud P. (2022). Course of post COVID-19 disease symptoms over time in the ComPaRe long COVID prospective e-cohort. *Nat. Commun.* 13:1812. 10.1038/s41467-022-29513-z 35383197 PMC8983754

[B96] Tröscher A. Gebetsroither P. Rindler M. Böhm V. Dormann R. von Oertzen T. (2024). High somatization rates, frequent spontaneous recovery, and a lack of organic biomarkers in post-COVID-19 condition. *Brain Behav.* 14:e70087. 10.1002/brb3.70087 39378280 PMC11460636

[B97] Tsampasian V. Elghazaly H. Chattopadhyay R. Debski M. Naing T. K. P. Garg P. (2023). Risk factors associated with post-COVID-19 condition: A systematic review and meta-analysis. *JAMA Intern. Med.* 183 566–580. 10.1001/jamainternmed.2023.0750 36951832 PMC10037203

[B98] van Kessel S. A. M. Olde Hartman T. C. Lucassen P. L. B. J. van Jaarsveld C. H. M. (2022). Post-acute and long-COVID-19 symptoms in patients with mild diseases: A systematic review. *Fam. Pract.* 39 159–167. 10.1093/fampra/cmab076 34268556 PMC8414057

[B99] Vanichkachorn G. Newcomb R. Cowl C. T. Murad M. H. Breeher L. Miller S. (2021). Post-COVID-19 syndrome (Long Haul Syndrome): Description of a multidisciplinary clinic at mayo clinic and characteristics of the initial patient cohort. *Mayo Clin. Proc.* 96 1782–1791. 10.1016/j.mayocp.2021.04.024 34218857 PMC8112396

[B100] Veronese N. Bonica R. Cotugno S. Tulone O. Camporeale M. Smith L. (2022). Interventions for improving long COVID-19 symptomatology: A systematic review. *Viruses* 14:1863. 10.3390/v14091863 36146672 PMC9502379

[B101] Walker S. Goodfellow H. Pookarnjanamorakot P. Murray E. Bindman J. Blandford A. (2023). Impact of fatigue as the primary determinant of functional limitations among patients with post-COVID-19 syndrome: A cross-sectional observational study. *BMJ Open* 13:e069217. 10.1136/bmjopen-2022-069217 37286327 PMC10335413

[B102] Wong M. C. Huang J. Wong Y. Y. Wong G. L. Yip T. C. Chan R. N. (2023). Epidemiology, symptomatology, and risk factors for long COVID symptoms: Population-based, multicenter study. *JMIR Public Health Surveill.* 9:e42315. 10.2196/42315 36645453 PMC9994465

[B103] World Health Organization [WHO] (2022). *Post COVID-19 condition (Long COVID).* Geneva: World Health Organization.

[B104] World Health Organization [WHO] (2024). *Number of COVID-19 cases reported to WHO.* Geneva: World Health Organization.

[B105] Yong S. J. (2021). Long COVID or post-COVID-19 syndrome: Putative pathophysiology, risk factors, and treatments. *Infect. Dis.* 53 737–754. 10.1080/23744235.2021.1924397 34024217 PMC8146298

[B106] Zhang Y. Romieu-Hernandez A. Boehmer T. K. Azziz-Baumgartner E. Carton T. W. Gundlapalli A. V. (2024). Association between SARS-CoV-2 infection and select symptoms and conditions 31 to 150 days after testing among children and adults. *BMC Infect. Dis.* 24:181. 10.1186/s12879-024-09076-8 38341566 PMC10859007

[B107] Zhou Y. Feng H. Li G. Xu C. Wu Y. Li H. (2022). Efficacy of computerized cognitive training on improving cognitive functions of stroke patients: A systematic review and meta-analysis of randomized controlled trials. *Int. J. Nurs. Pract.* 28:e12966. 10.1111/ijn.12966 34036682

[B108] Ziauddeen N. Gurdasani D. O’Hara M. E. Hastie C. Roderick P. Yao G. (2022). Characteristics and impact of Long COVID: Findings from an online survey. *PLoS One* 17:e0264331. 10.1371/journal.pone.0264331 35259179 PMC8903286

