# Supplementary material for: A tablet-based intervention study to alleviate cognitive and psychological symptoms in patients with post-Covid-19 condition
Source: Front Psychol. 2025 Aug 25;16:1582742. doi: 10.3389/fpsyg.2025.1582742 (PMC12414737; doi:10.3389/fpsyg.2025.1582742)
Supplement: Supplementary file 1 [file Table_1.docx]

**Supplement Table 1**. Acute and persistent symptoms of post-Covid-19 patients during baseline (BL), follow-up 1 (FU1) and follow-up 2 (FU2).

| **Symptom** | **Acute phase** | | **Ongoing phase** | | | | | |
| --- | --- | --- | --- | --- | --- | --- | --- | --- |
|  |  | | *BL* | | *FU1* | | *FU2* | |
|  | n | % | n | % | n | % | n | % |
| Fatigue | 25 | 62.5 | 16 | 40.0 | 24 | 60.0 | 16 | 40.0 |
| Exhaustion | 29 | 72.5 | 15 | 37.5 | 18 | 45.0 | 14 | 35.0 |
| Memory impairment | 3 | 7.5 | 34 | 85.0 | 26 | 65.0 | 21 | 52.5 |
| Brain fog | 6 | 15.0 | 35 | 87.5 | 15 | 37.5 | 13 | 32.5 |
| Dyspnea | 20 | 50.0 | 8 | 20.0 | 14 | 35.0 | 6 | 15.0 |
| Discomfort after exertion | 8 | 20.0 | 3 | 7.5 | 11 | 27.5 | 8 | 20.0 |
| Chest tightness | 7 | 17.5 | 5 | 12.5 | 7 | 17.5 | 3 | 7.5 |
| Dizziness | 12 | 30.0 | 5 | 12.5 | 7 | 17.5 | 5 | 12.5 |
| Headache | 21 | 52.5 | 8 | 20.0 | 8 | 20.0 | 7 | 17.5 |
| Insomnia | 8 | 20.0 | 6 | 15.0 | 10 | 25.0 | 9 | 22.5 |
| Difficulty breathing | 11 | 27.5 | 4 | 10.0 | 7 | 17.5 | 4 | 10.0 |
| Muscle pain | 17 | 42.5 | 11 | 27.5 | 8 | 20.0 | 8 | 20.0 |
| Altered taste/smell | 25 | 62.5 | 8 | 20.0 | 9 | 22.5 | 6 | 15.0 |
| Heart palpitations | 4 | 10.0 | 2 | 5.0 | 2 | 5.0 | 1 | 2.5 |
| Joint pain | 13 | 32.5 | 7 | 17.5 | 7 | 17.5 | 5 | 12.5 |
| Dry cough | 18 | 45.0 | 1 | 2.5 | 4 | 10.0 | 4 | 10.0 |
| Chills / sweating | 13 | 32.5 | 5 | 12.5 | 5 | 12.5 | 5 | 12.5 |
| Other sleep symptoms | 3 | 7.5 | 1 | 2.5 | 4 | 10.0 | 6 | 15.0 |
| Nausea | 6 | 15.0 | 0 | 0.0 | 3 | 7.5 | 0 | 0.0 |
| Diarrhea | 8 | 20.0 | 3 | 7.5 | 1 | 2.5 | 3 | 7.5 |
| Tremor | 3 | 7.5 | 1 | 2.5 | 1 | 2.5 | 2 | 5.0 |
| Slurred speech | 1 | 2.5 | 0 | 0.0 | 0 | 0.0 | 0 | 0.0 |
| Tinnitus | 0 | 0.0 | 3 | 7.5 | 6 | 15.0 | 5 | 12.5 |
| Fever | 19 | 47.5 | 0 | 0.0 | 2 | 5.0 | 0 | 0.0 |
| Stomach ache | 3 | 7.5 | 0 | 0.0 | 0 | 0.0 | 1 | 2.5 |
| Elevated body temperature (37-38°C) | 4 | 10.0 | 1 | 2.5 | 0 | 0.0 | 0 | 0.0 |
| Skin abnormalities / allergies | 2 | 5.0 | 3 | 7.5 | 3 | 7.5 | 3 | 7.5 |
| Heartburn / reflux | 6 | 15.0 | 6 | 15.0 | 6 | 15.0 | 4 | 10.0 |
| Weight loss | 2 | 5.0 | 0 | 0.0 | 0 | 0.0 | 0 | 0.0 |
| Bladder control issues | 0 | 0.0 | 2 | 5.0 | 3 | 7.5 | 1 | 2.5 |
| Menstrual irregularities | 1 | 2.5 | 2 | 5.0 | 0 | 0.0 | 2 | 5.0 |
| Skin rash | 1 | 2.5 | 1 | 2.5 | 2 | 5.0 | 1 | 2.5 |
| Constipation | 0 | 0.0 | 1 | 2.5 | 0 | 0.0 | 0 | 0.0 |
| Decreased body temperature | 1 | 2.5 | 0 | 0.0 | 2 | 5.0 | 0 | 0.0 |
| Emesis | 4 | 10.0 | 0 | 0.0 | 0 | 0.0 | 0 | 0.0 |
| Hearing impairment | 0 | 0.0 | 1 | 2.5 | 0 | 0.0 | 1 | 2.5 |
| Disorientation / confusion | 2 | 5.0 | 2 | 5.0 | 1 | 2.5 | 2 | 5.0 |
| Loss of appetite | 9 | 22.5 | 0 | 0.0 | 1 | 2.5 | 0 | 0.0 |
| Sore throat | 20 | 50.0 | 1 | 2.5 | 3 | 7.5 | 1 | 2.5 |
| Runny nose | 4 | 10.0 | 0 | 0.0 | 5 | 12.5 | 5 | 12.5 |
| Disturbed neurological sensation | 2 | 5.0 | 0 | 0.0 | 3 | 7.5 | 4 | 10.0 |
| Burning chest pain | 1 | 2.5 | 1 | 2.5 | 0 | 0.0 | 0 | 0.0 |
| Tachycardia | 3 | 7.5 | 2 | 5.0 | 2 | 5.0 | 2 | 5.0 |
| Sneezing | 3 | 7.5 | 0 | 0.0 | 2 | 5.0 | 0 | 0.0 |
| Visual disturbances | 1 | 2.5 | 5 | 12.5 | 3 | 7.5 | 3 | 7.5 |
| Bone pain | 2 | 5.0 | 0 | 0.0 | 0 | 0.0 | 1 | 2.5 |
| Mucus cough | 6 | 15.0 | 0 | 0.0 | 2 | 5.0 | 1 | 2.5 |
| Nerve pain | 3 | 7.5 | 2 | 5.0 | 5 | 12.5 | 3 | 7.5 |
| New allergies | 1 | 2.5 | 0 | 0.0 | 0 | 0.0 | 2 | 5.0 |
| Hallucinations | 1 | 2.5 | 0 | 0.0 | 0 | 0.0 | 0 | 0.0 |
| Dermographism | 1 | 2.5 | 0 | 0.0 | 0 | 0.0 | 0 | 0.0 |
| Bradycardia | 1 | 2.5 | 0 | 0.0 | 1 | 2.5 | 0 | 0.0 |
| Muscle cramps | 3 | 7.5 | 3 | 7.5 | 0 | 0.0 | 4 | 10.0 |
| Other symptoms ^a^ | 19 | 47.5 | 18 | 45.0 | 0 | 0.0 | 0 | 0.0 |

*Note*. ^a^ Other acute symptoms: dry nose/throat, eye pain, sensitivity to light, bronchitis, heightened blood pressure, nosebleed, digestive problems, heart pain. Other post-Covid symptoms (BL): asthma, trouble finding words, depression, hair loss, numbness, sensitivity to light, fecal incontinence, swap words, susceptibility to infections, neck pain, difficulty with reading comprehension, limited multitasking ability.
