# Supplementary material for: A tablet-based intervention study to alleviate cognitive and psychological symptoms in patients with post-Covid-19 condition
Source: Front Psychol. 2025 Aug 25;16:1582742. doi: 10.3389/fpsyg.2025.1582742 (PMC12414737; doi:10.3389/fpsyg.2025.1582742)
Supplement: Supplementary file 2 [file Table_2.docx]

**Supplement Table 2**. Results of the 2x3 ANCOVAs. Presented is the significance of the group (2 levels: control group, intervention group) x time (3 levels: BL, FU1, FU2) interaction effect to evaluate the efficacy of a three-month tablet-based training program for post-Covid-19 patients.

| **Domain** | **Subtest** | ***n*** | ***F*** | **df** | **Error df** | ***p*** | $\boldsymbol{\eta}_{\boldsymbol{p}}^{\mathbf{2}}$ |
| --- | --- | --- | --- | --- | --- | --- | --- |
| **Attention** | Digit span forward^a^ | 30 | 6.11 | 2 | 50 | **.004** | .196 |
|  | Digit span backward^a^ | 30 | 0.85 | 2 | 50 | .434 | .033 |
|  | TMT-A [time]^b^ | 30 | 1.95 | 2 | 52 | .152 | .070 |
| **Executive function** | Planning^c^ | 30 | 1.94 | 2 | 52 | .154 | .069 |
|  | Categories^a^ | 30 | 2.04 | 2 | 50 | .140 | .076 |
|  | TMT-B [time]^a^ | 30 | 1.16 | 2 | 50 | .321 | .044 |
| **Memory** | Immediate recall (A)^a^ | 30 | 0.28 | 1.53 | 38.36 | .700 | .011 |
|  | Immediate recall (B)^a^ | 30 | 1.88 | 2 | 50 | .164 | .070 |
|  | Short-delayed recall^b^ | 30 | 0.02 | 2 | 52 | .981 | .001 |
|  | Long-delayed recall^b^ | 30 | 0.35 | 1.56 | 40.57 | .657 | .013 |
| **Word fluency** | Formal-lexical^b^ | 30 | 0.34 | 2 | 52 | .713 | .013 |
|  | Semantic^c^ | 30 | 0.71 | 2 | 52 | .497 | .027 |
| **Global cognition** | MoCA^c^ | 30 | 2.44 | 2 | 52 | .097 | .086 |
|  | MoCA memory index^a^ | 30 | 2.25 | 2 | 50 | .116 | .083 |
| **Subjective Cognitive Complaints** | FSKB [QPC]^a^ | 30 | 2.11 | 2 | 50 | .132 | .078 |
| **Fatigue** | FISD total^b^ | 27 | 1.86 | 2 | 46 | .168 | .075 |
| **Depression** | ADS^a^ | 30 | 2.01 | 2 | 50 | .145 | .074 |
|  | HADS-D^c^ | 30 | 0.96 | 2 | 52 | .391 | .036 |
| **Anxiety** | HADS-A^a^ | 30 | 0.25 | 2 | 50 | .780 | .010 |
| **Quality of Life** | WHOQoL-Physical^a^ | 30 | 1.82 | 1.45 | 36.18 | .184 | .068 |
|  | WHOQoL-Psychological^a^ | 30 | 1.92 | 2 | 50 | .157 | .071 |

*Note*. ^a^all covariates included as assumptions were met, ^b^ sex excluded as a covariate, ^c^ education excluded as a covariate. Significant results are highlighted in bold.
